# An ultra-short-period super-Earth with an extremely high density and an outer companion

**DOI:** 10.1038/s41598-024-76490-y

**Published:** 2024-11-08

**Authors:** John H. Livingston, Davide Gandolfi, Alessandro A. Trani, Mahesh Herath, Oscar Barragán, Artie Hatzes, Rafael Luque, Akihiko Fukui, Grzegorz Nowak, Enric Palle, Coel Hellier, Malcolm Fridlund, Jerome de Leon, Teruyuki Hirano, Norio Narita, Simon Albrecht, Fei Dai, Hans Deeg, Vincent Van Eylen, Judith Korth, Motohide Tamura

**Affiliations:** 1 Astrobiology Center, NINS, 2-21-1 Osawa, Mitaka, Tokyo 181-8588 Japan; 2 National Astronomical Observatory of Japan, NINS, 2-21-1 Osawa, Mitaka, Tokyo 181-8588 Japan; 3 Astronomical Science Program, Graduate University for Advanced Studies, SOKENDAI, 2-21-1, Osawa, Mitaka, Tokyo 181-8588 Japan; 4 Dipartimento di Fisica, Universitá di Torino, via P. Giuria 1, 10125 Turin, Italy; 5 Niels Bohr International Academy, Niels Bohr Institute, Blegdamsvej 17, 2100 Copenhagen, Denmark; 6 School of Science, Research Center for the Early Universe, The University of Tokyo, Tokyo, 113-0033 Japan; 7 Okinawa Institute of Science and Technology, 1919-1 Tancha, Onna-son, Okinawa 904-0495 Japan; 8 Trottier Space Institute, McGill University, Montreal, QC H3A 2A7 Canada; 9 Department of Earth and Planetary Science, McGill University, 3450 Rue University, Montreal, QC H3A 0E8 Canada; 10 Department of Physics, Sub-department of Astrophysics, University of Oxford, Oxford, OX1 3RH UK; 11 Thüringer Landessternwarte Tautenburg, Sternwarte 5, 07778 Tautenberg, Germany; 12 Department of Astronomy and Astrophysics, University of Chicago, Chicago, IL 60637 USA; 13 Komaba Institute for Science, The University of Tokyo, 3-8-1 Komaba, Meguro, Tokyo 153-8902 Japan; 14 Faculty of Physics, Astronomy and Informatics, Institute of Astronomy, Nicolaus Copernicus University, Grudzia̧dzka 5, 87-100 Toruń, Poland; 15 Instituto de Astrofísica de Canarias, C. Vía Láctea S/N, 38205 La Laguna, Tenerife Spain; 16 Dept. de Astrofísica, Universidad de La Laguna, 38206 La Laguna, Tenerife Spain; 17 Astrophysics Group, Keele University, Staffordshire, ST5 5BG UK; 18 Department of Space, Earth and Environment, Chalmers University of Technology, Onsala Space Observatory, 439 92 Onsala, Sweden; 19 Department of Physics and Astronomy, Stellar Astrophysics Centre, Aarhus University, Ny Munkegade 120, 8000 Aarhus C, Denmark; 20 Institute for Astronomy, University of Hawai’i, 2680 Woodlawn Drive, Honolulu, HI 96822 USA; 21 Division of Geological and Planetary Sciences, 1200 E California Blvd, Pasadena, CA 91125 USA; 22 Department of Astronomy, California Institute of Technology, Pasadena, CA 91125 USA; 23 Mullard Space Science Laboratory, University College London, Holmbury St Mary, Dorking, Surrey RH5 6NT UK; 24 Lund Observatory, Division of Astrophysics, Department of Physics, Lund University, Box 118, 22100 Lund, Sweden; 25 Department of Astronomy, The University of Tokyo, 7-3-1 Hongo, Bunkyo-ku, Tokyo 113-0033 Japan

**Keywords:** Planetary science, Exoplanets

## Abstract

We present the discovery and characterization of a new multi-planetary system around the Sun-like star K2-360 (EPIC 201595106). K2-360 was first identified in *K2* photometry as the host of an ultra-short-period (USP) planet candidate with a period of 0.88 d. We obtained follow-up transit photometry, confirming the star as the host of the signal. High precision radial velocity measurements from HARPS and HARPS-N confirm the transiting USP planet and reveal the existence of an outer (non-transiting) planet with an orbital period of $\sim$10 d. We measure a mass of $7.67 \pm 0.75$ $M_{\oplus }$ and a radius of $1.57 \pm 0.08$ $R_{\oplus }$ for the transiting planet, yielding a high mean density of $11 \pm 2$ g $\hbox {cm}^{-3}$, making it the densest well-characterized USP super-Earth discovered to date. We measure a minimum mass of $15.2 \pm 1.8$ $M_{\oplus }$ for the outer planet, and explore a migration formation pathway via the von Zeipel–Kozai–Lidov (ZKL) mechanism and tidal dissipation.

## Introduction

As first illuminated by the *Kepler* mission^1^, planets with sizes and orbits unrepresented in the Solar system are common, and many of them present challenges for current theories of formation and evolution. Planets with orbital periods shorter than one day, also known as ultra-short-period planets (USPs)^2,3^, are an emergent class of planet that has hitherto eluded explanation by current theory. The dynamical history and evolution of USPs are of particular interest^4^, but to date few observational studies have probed this in detail^5,6^. From the earliest *Kepler* discoveries to more recent findings of the Transiting Exoplanet Survey Satellite (*TESS*) mission^7^, USPs represent a distinct and growing population of poorly understood planets that tend to have high densities and mutual inclinations^8–10^. In this work, we focus on small, rocky USPs ($R_P < 2 R_\oplus$, $M_P < 12 M_\oplus$), as opposed to larger, more massive USPs with substantial gaseous envelopes^11^, which potentially have a separate formation pathway. As in the case of hot Jupiters (HJs), USPs cannot have formed in their present location. Furthermore, there is reason to believe USPs are not simply the highly irradiated cores of HJs^12^, thus requiring an entirely separate formation pathway. These planets must therefore have formed further out in the system and migrated inwards, e.g. via Type I disk migration or dynamical interaction with other planets after disk dispersal, but to date little is known about the particular pathway. One possible path towards a better understanding of USP formation is thus to search for clues about the dynamical history of USPs in multi-planet systems, as their present-day architectures and physical characteristics contain an imprint of the dominant mechanisms at play in the systems’ past. The expectation for a migration history involving the von Zeipel–Kozai–Lidov (ZKL) mechanism and tidal dissipation is the presence of a more massive non-coplanar outer planet.

In this work, we report the discovery and characterization of a multi-planet system around the Sun-like star K2-360 (EPIC 201595106), using photometry from *K2*, the extended *Kepler* mission, and high precision radial velocity (RV) measurements from HARPS and HARPS-N. The system contains both a transiting USP and a non-transiting outer planet, for which we constrain masses and eccentricities via detailed analyses of the photometry and RV data. K2-360 was first reported to host a candidate USP planet with a low false positive probability (FPP) of 0.2%^13^, but it was not validated due to an abundance of caution regarding a nearby star contributing flux to the photometric aperture, which could potentially have been the source of the observed transit signal. The candidate was subsequently validated in a catalog of *K2* USPs^14^; however, the signal was assumed to be on-target and no additional observations or analyses were conducted to confirm this to be the case. We present follow-up transit photometry that confirms K2-360 as the host of the transit signal, and high precision RV measurements from HARPS and HARPS-N that independently confirm the planetary nature of the USP and measure its mass. The RV data also reveal the existence of a second planet with a longer period, which does not transit the host star. Interior structure modeling, as well as dynamical N-body and tidal simulations, reveal the nature of the system in striking detail and provide important clues as to its formation.

## Results

### Origin of the transit signal

**Fig. 1:**
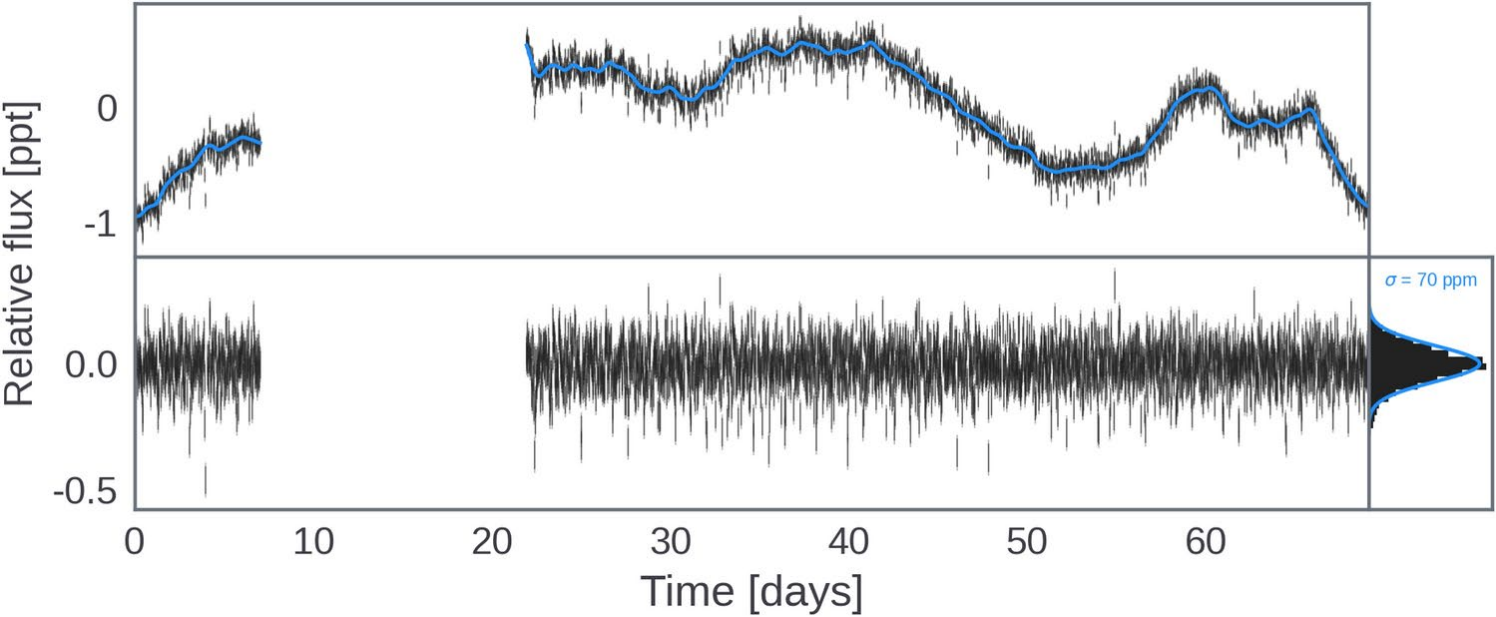
*K2* light curve (gray points) with GP variability model (top); time series residuals with marginal histogram and the total noise estimate from the model (bottom). Note the transits are not easily discernible by eye, but can be seen as a heavy tail in the marginal histogram.

**Fig. 2:**
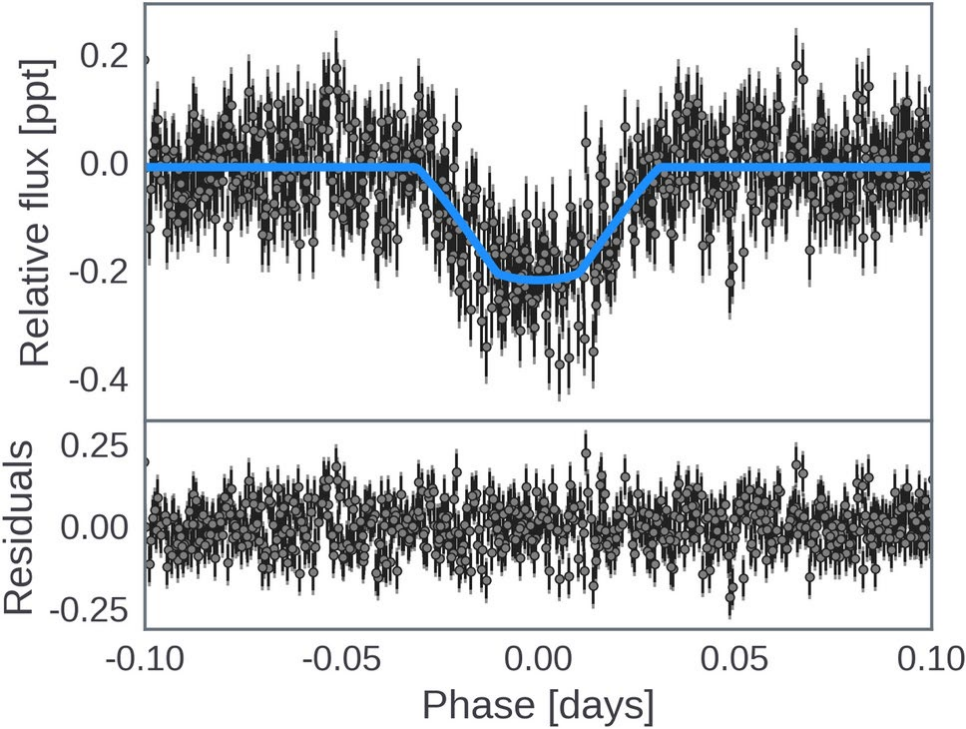
Flattened and phase-folded *K2* light curve with best-fitting transit model overplotted in blue (top), and residuals (bottom).

K2-360 was observed during Campaign 10 of the *K2* mission^15^ from 2016 July 6 to September 20, using channel 70 (module 20) of the *Kepler* photometer. However, a large pointing error in the initial 6 days and a subsequent failure of module 4 led to data loss. To correct for systematic noise induced by the spacecraft’s roll, we utilized the EVEREST package^16,17^ to produce a corrected *K2* light curve. This package selects an optimal photometric aperture and computes a systematics model based on pixel level decorrelation^18^. After masking out transits, the resulting light curve revealed stellar variability at the 1 ppt level, and after modeling and subtracting this variability the photometry had a precision of 70 ppm (Fig. 1). The previously reported Ultra-Short Period (USP) candidate was recovered from the *K2* light curve using the Box-Least-Squares (BLS) algorithm^19^ after modeling and subtracting stellar variability with a Gaussian process (GP) model^20^; subsequent searches to find additional transiting planets using BLS yielded no further signals. However, due to the shallow transit depth, *Kepler* ’s pixel scale of 4”, and the size of the photometric aperture, contamination from a nearby star at 13.6” (EPIC 201595004) had not been sufficiently ruled out, leaving room for doubt as to the true origin and nature of the observed signal. K2-360 was also observed by *TESS* camera 4, CCD 1, during Sector 46 (2021 December 2-30), resulting in 22.6 days of photometry with no quality flags. Given its orbital period, $\sim$26 transits of K2-360 b should be present in the data, but the photometric precision is too low (2.1 ppt RMS per 2 minutes) to detect the 0.2 ppt transits of K2-360 b (the phase-folded SNR is $\sim$2.7). Furthermore, the pixel scale of *TESS* is even larger than *Kepler*, so it is unable to help in this regard. We therefore conducted additional observations to confirm or rule out an on-target source for the signal.

On the night of 2018 February 23 UT, K2-360 was observed with MuSCAT^21^, a simultaneous multi-band imager on the 1.88 m telescope in Okayama, Japan. The achieved photometric precision was insufficient to detect the shallow transit of K2-360 b, but was sufficient to rule out the possibility of the transit signal originating from the nearby star (EPIC 201595004). Thus, by confirming the transit signal to be on-target, and given the false positive probability (FPP) of 0.2%^13^, the MuSCAT photometry enabled validation of the planet at 99.8% confidence. On UT 2017 March 18, observations of K2-360 were conducted using the NASA Exoplanet Star and Speckle Imager (NESSI) instrument^22,23^ mounted on the 3.5 m WIYN telescope. NESSI utilizes high-speed electron-multiplying CCDs to capture sequences of 40 ms exposures in blue and red bands simultaneously. No secondary sources were detected, and background sensitivity was measured using concentric annuli centered on the target star. The NESSI data achieve a contrast of $\Delta$mag$\sim$4-5 (5$\sigma$) at separations of $\sim$0.2-1”, further reducing the potential of a source for the transit signal other than K2-360 from a statistical improbability to a near impossibility.

### Characterization of the host star

We derived stellar parameters from combined HARPS and HARPS-N spectra using the SME analysis framework^24^, with fixed macroscopic and microscopic turbulence velocities^25,26^. We obtained effective temperature, surface gravity, calcium abundance, iron abundance, and projected rotational velocity from both the the HARPS and HARPS-N data, and computed the weighted mean of these values. We then inferred a full set of stellar parameters using the isochrones package^27^ and the MIST stellar model grid^28^, relying on optical and near-infrared photometry from Gaia, 2MASS, and WISE, as well as the star’s parallax. We also used the astroARIADNE package^29^ to estimate stellar parameters with a Bayesian Model Averaging scheme; these were in good agreement, suggesting robust parameter estimates unlikely to suffer significantly from biases due to model dependence. The host star was found to be Sun-like, with an effective temperature of $T_{\textrm{eff}}$  = $5679^{+60}_{-39}$ K and an age of $6.0^{+2.6}_{-2.9}$ Gyr.

We conducted an analysis of the *K2* light curve for rotation-induced stellar spot modulation using the Generalized Lomb-Scargle (GLS) periodogram^30^ and the auto-correlation function (ACF). Considering both methods together, the *K2* photometry suggests a rotation period ($P_\textrm{rot}$) in the range of $\sim$20–40 days. The field of K2-360 was also observed by WASP-South during the WASP transit survey^31^ in 2009 and 2010. Analysis of the WASP datasets^32^ revealed significant $\sim$3 mmag periodicities, with the combined dataset showing significant power in the 26–30 day range, consistent with $v \sin i$ within uncertainties. The period was determined to be 30 ± 2 days and 26 ± 2 days for 2009 and 2010, respectively. Analysis of nearby stars showed no similar periodicity, ruling out moonlight contamination. Analyses of spectroscopic activity indicators revealed significant peaks at approximately 15 and 28 days, suggesting a true rotation period of around 28 days and a starspot configuration that produces power at both the period and the first harmonic^33,34^. We adopted the weighted mean of the two WASP constraints as an estimate for $P_\textrm{rot}$, with a value of $28.0 \pm 1.4$ d, but we note that this uncertainty may be significantly underestimated.

### Light curve modeling

We conducted a stellar variability fit to the *K2* data employing the exoplanet^35^ and PyMC3^36^ packages. The variability was modeled using a simple harmonic oscillator (SHO) kernel to represent stochastically-driven, damped harmonic oscillations. Hyperparameters were explored in log space with wide Gaussian priors, and a jitter term was added to prevent overfitting. Prior to fitting the GP, transits were masked out based on twice the transit duration determined by the Box-Least-Squares (BLS) search. Maximum a posteriori (MAP) parameter estimates were obtained using the BFGS algorithm^37^, and the resulting light curve, along with the best-fit GP model, residuals, and a histogram comparing residuals to total noise, were visualized in Fig. 1.

Subsequently, a transit fit was performed on the *K2* data after removing the GP variability model. This analysis also utilized the exoplanet and PyMC3 frameworks for inference, with the Starry package^38^ used for the transit model. Various parameters, including a bias term, white noise scale factor, and limb darkening, were included in the model. Initially, a MAP estimate was used to identify and remove 5$\sigma$ outliers before the model was refitted. Hamiltonian Monte Carlo (HMC) sampling via the NUTS algorithm^39^ was then initialized, and the Gelman-Rubin statistic^40^ confirmed well-mixed sampling. We found a planet-to-star radius ratio of $0.0147 \pm 0.0007$, yielding radius measurement of 1.57±0.08 $R_{\oplus }$. The phase-folded photometry with the best-fitting transit model is shown in Fig. 2.

### Doppler mass measurement

**Fig. 3:**
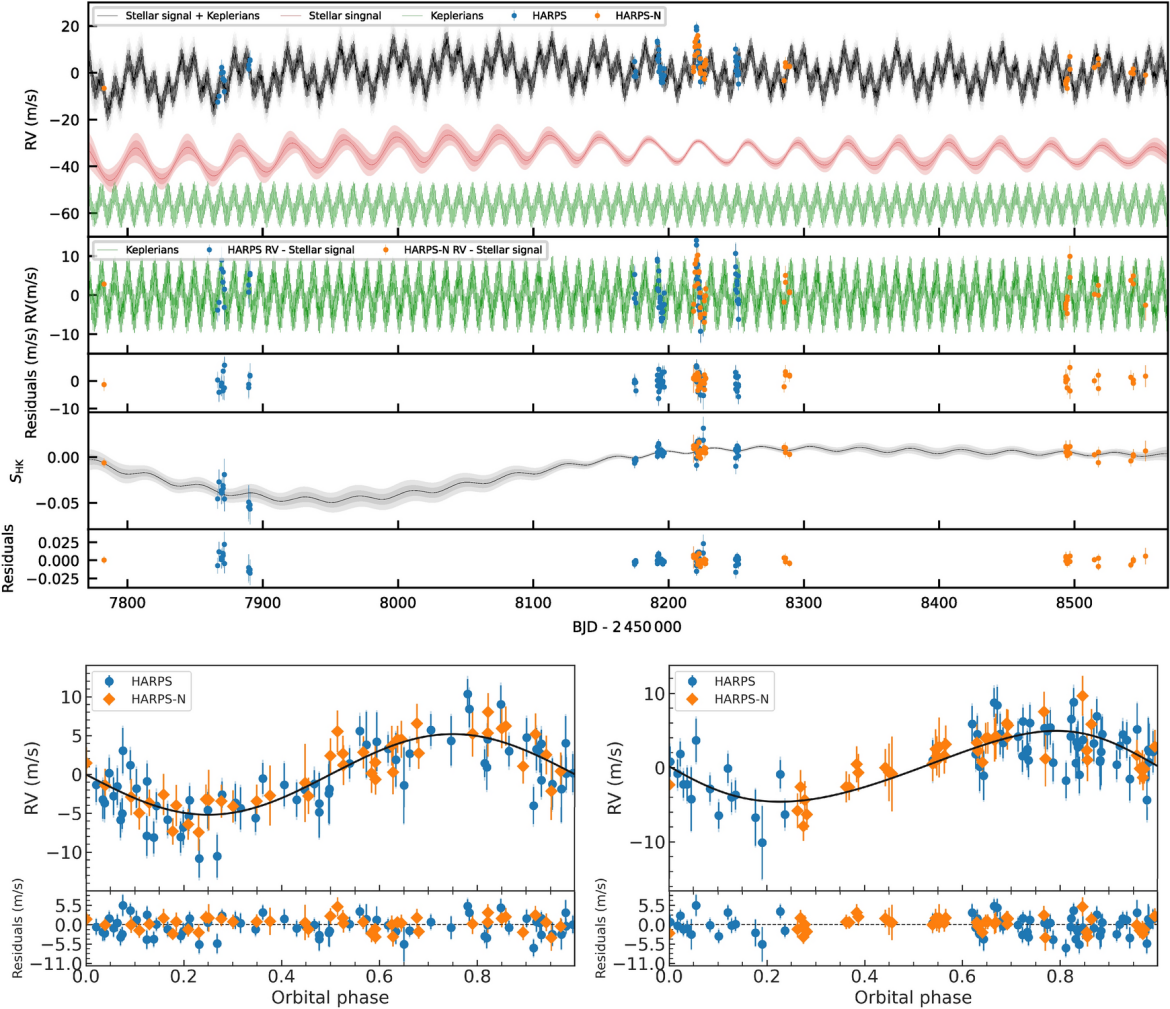
*Upper panels*—RV and $S_\textrm{HK}$ time series. Each plot shows (from top to bottom): RV data together with full (stellar plus planetary signal inferred models); RV data with stellar signal model subtracted; RV residuals; $S_\textrm{HK}$ data together with inferred stellar model, and $S_\textrm{HK}$ residuals. All time series are shown after been corrected by inferred offsets. For the RV time series we also show the inferred stellar (red line) and planetary (green line) recovered signals with an offset to make them clearly visible. HARPS (blue) and HARPS-N (orange) RV and $S_\textrm{HK}$ are shown in the corresponding panels. Measurements are shown with filled symbols with error bars with a semi-transparent error bar extension accounting for the inferred jitter. The solid (black) lines show the inferred full model coming from our multi-GP, light grey shaded areas showing the one and two sigma credible intervals of the corresponding GP model. *Lower panels*—Phase-folded RV data for planets b (left) and c (right) with best-fit Keplerian model, after subtracting the RV signal of the other planet and stellar activity.

Spectroscopic follow-up of K2-360 was conducted using the HARPS spectrograph^41^ mounted on the ESO-3.6 m telescope and the HARPS-N spectrograph^42^ on the 3.58 m Telescopio Nazionale Galileo (TNG). Radial velocity measurements and activity indicators were obtained using DRS^43^ and SERVAL^44^, providing crucial data for the study of the K2-360 system.

In order to accurately determine a planetary mass from radial velocities (RVs), it is crucial to investigate potential signals from stellar activity and additional massive bodies in the system. Frequency analyses were conducted on the RVs and activity indicators from both pipelines, considering instrumental offsets between HARPS and HARPS-N to ensure accurate combination of time series measurements. GLS periodograms were computed for the combined HARPS and HARPS-N RV measurements and activity indicators from both pipelines, revealing a clear peak in the RV periodogram at the orbital period of the USP planet and another prominent peak at  9.6 days, suggesting a Doppler detection of the USP and a possible second planet. Significant peaks were seen at 15, 28, 62, and 79 days in the activitiy indicators; given the modulation seen in the *K2* and WASP light curves, along with the expected rotation period implied by $v \sin i$ and the stellar radius, the shorter period signals are likely related to stellar rotation, while the longer-period signals may be related to spot evolution^45^. The lack of any counterparts for the 9.6 day RV signal in the activity indicators suggests it is not caused by stellar chromospheric activity, and therefore a planetary origin is more likely. Additionally, we computed $\ell _1$ periodograms of the RVs, leveraging a publicly available code^46,47^ known for its effectiveness in recovering sparse signals in the presence of noise. The $\ell _1$ periodogram corroborated the USP and 9.6 day signals, but revealed a third signal at $\sim$38 days instead of $\sim$30 days. This discrepancy reflects the ambiguity of the rotation signal in the RVs, most likely due to the weakness of the signal, decoherence of the spot configuration on the timescale of the observations, and/or differential rotation^48^. We note that the HARPS data in particular show clear peaks in the GLS periodograms of both the BIS and S-index ($S_\textrm{HK}$) activity indicators near the adopted rotation period ($\sim$28 days) and its first harmonic, suggesting the rotation signal may be stronger in the indicators than in the RVs themselves. We also computed Stacked Bayesian GLS periodograms (SBGLS) of the RVs^49^, which reveal the effect of data quantity on signal strength and coherence. The SBGLS results further reinforce the interpretation that the USP and 9.6 day signals are coherent and planetary in nature, in contrast with the longer period signals, which are less coherent due to the more variable nature of stellar activity.

The RV measurements of K2-360 include up to four measurements per night, covering about 20% of the transiting planet’s orbital curve. This dataset lends itself well to the “floating chunk offset” (FCO) method^50^, where data is divided into time chunks spanning several hours to fit an orbit while allowing the zero point offset in each chunk to vary. This approach naturally combines different data sets and accounts for systematic errors, assuming variations longer than the length of the time chunks. By applying the FCO method the reduced $\chi ^2$ is minimized at the period of the transiting USP planet, indicating a real signal at this period. Besides the agreement with the transit period, the semi-amplitude of the planet is detected to better than 5$\sigma$ significance. The FCO method clearly recovered the transit ephemeris, but to ensure that a false signal was not somehow introduced by the method, simulated data sets with periodic signals were analyzed, which confirmed FCO’s accuracy in recovering the input amplitudes. The GLS periodogram of the RV residuals after removing the contribution of the transiting planet showed a significant peak at $\approx$ 0.1 $\hbox {d}^{-1}$, with a false alarm probability (FAP) less than 5$\times 10^{-6}$, corroborating the signal found by the other methods. Given the lack of signals at this period in the activity indicators, the most plausible explanation for these RV variations is the presence of an additional, non-transiting planet, K2-360  c.

To better understand the nature of the stellar activity, we utilized GP regression to analyze our activity indicator time series and determine the GP hyperparameters defining the stellar signal characteristics. Each activity indicator time series was modeled using the covariance function, where A represents the amplitude term, and $\gamma _{i,j}$ is the Quasi-Periodic (QP) kernel defined by the GP period ($P_\textrm{GP}$), the inverse of the harmonic complexity ($\lambda _p$), and the long term evolution timescale ($\lambda _e$). To account for instrumental imperfections, we included offset and jitter terms per instrument in the likelihood function. The posterior distributions of the QP kernel hyperparameters were computed using pyaneti^51,52^. We conducted one-dimensional GP regression for the full width at half-maximum (FWHM) of the cross-correlation function and the S-index to characterize the scales of the stellar-induced signals, setting wide uniform priors on the GP hyperparameters. The parameter space was sampled using 250 walkers with the Markov chain Monte Carlo (MCMC) ensemble sampler implemented in pyaneti^51,53^, resulting in posterior distributions for the QP hyperparameters of $\lambda _\textrm{e} = 383 _{ - 147 } ^ { + 85 }$ days, $\lambda _\textrm{p} = 3.5 _{ - 2.1 } ^ { + 3.6 }$, and $P_\textrm{GP} = 34.8 _{ - 1.1 } ^ { + 1.0 }$ days from the FWHM data. Similarly, for the $S_\textrm{HK}$ time series, the recovered QP hyperparameters were $\lambda _\textrm{e} = 157 _{ - 50 } ^ { + 59 }$ days, $\lambda _\textrm{p} = 6.8 _{ - 2.7 } ^ { + 2.2 }$, $P_\textrm{GP} = 31.8 _{ - 2.9 } ^ { + 6.7 }$ days. These results exhibited some inconsistency, possibly due to sub-optimal sampling of the spectroscopic time series, but they are supportive of the plausible rotation signals found by frequency analysis. The low harmonic complexity inferred from both time series suggests the RVs should also exhibit a low-harmonic complexity^52^.

To assess signal significance and select an appropriate model for the RVs^54^, we employed a Bayesian log-evidence calculation using the juliet code^55^. Models were considered to be favored over others if their log-evidence difference $\Delta \ln Z$ exceeded 2.5^56^. We considered several RV-only models, varying from circular to eccentric orbits with increasing Keplerian components. To model stellar activity, we tested sinusoidal and quasi-periodic Gaussian Process kernel approaches, with GP hyperparameters informed by a GP analysis of the *K2* light curve. The top-performing model comprised a circular orbit for the USP, an eccentric orbit for the non-transiting planet, and a GP noise model with a quasi-periodic kernel. While modeling the stellar component with a sinusoid improved over two-component models, it failed to adequately capture observed stellar variability changes over time, resulting in a preference for the use of a GP model. This is likely due to the multi-year RV dataset encompassing different stellar activity regimes, resulting in decoherence of the rotation signal, as found by the SBGLS periogogram. The preference for an eccentric orbit for the non-transiting planet is also consistent with our FCO analysis results.

To obtain a final set of dynamical masses and orbital parameter estimates, we conducted a multidimensional GP analysis^57^ of the spectroscopic time series data using the pyaneti software package^51,52^. A 2-dimensional GP approach was adopted between the RVs and the $S_\textrm{HK}$due to the latter tracing stellar variability^58,59^. We assumed that the RV and $S_\textrm{HK}$ time series can be modeled as functions of a latent variable (*G*(*t*)) representing the projected area of the visible stellar disc covered in active regions at a given time. A covariance matrix was created using a quasi-periodic (QP) kernel, and posterior sampling was performed via an MCMC sampling method. The results of the GP analysis revealed Doppler semi-amplitudes of $5.19 \pm 0.51$ $\textrm{m}\,\textrm{s}^{-1}$ and $4.77 \pm 0.71$ $\textrm{m}\,\textrm{s}^{-1}$ for planets b and c, corresponding to planetary masses of $7.7 \pm 0.8 M_\oplus$ and $15.2 \pm 1.8 M_\oplus$, respectively. A small eccentricity of $0.11_{-0.08}^{+0.10}$ was recovered for planet c, but we note that this value is consistent with a circular orbit. The inferred values for the QP hyperparameters were $\lambda _\textrm{e} = 206_{-51}^{+71}$ days, $\lambda _\textrm{p} = 7.5_{-2.2}^{+1.8}$, and $P_\textrm{GP} = 37.6_{-1.1}^{+0.7}$ days. The relatively high value of $\lambda _\textrm{p}$ suggests a low harmonic complexity in the stellar signal, as expected from the GP analysis of the activity indicators. The data and models are depicted in Fig. 3, and the parameter estimates are tabulated in Table 1.

**Table 1:** Planet and miscellaneous model parameters.

Parameter	Unit	Planet b	Planet c
Planet parameters			
*P*	days	$0.87724 \pm 0.00004$	$9.79692 \pm 0.02961$
$T_0$	BKJD	$2750.04429 \pm 0.00157$	$3371.26490 \pm 0.32$
$R_P/R_\star$	$\cdots$	$0.0147 \pm 0.0007$	$\cdots$
*b*	$\cdots$	$0.814 \pm 0.024$	$\cdots$
*e*	$\cdots$	$\equiv 0$	$0.11^{+0.10}_{-0.08}$
$\omega$	deg.	$\equiv 0$	$71^{+87}_{-225}$
$R_P$	$R_\oplus$	$1.565 \pm 0.079$	$\cdots$
$M_P$	$M_\oplus$	$7.67 \pm 0.75$	$15.23 \pm 1.82^a$
$\rho _P$	g $\hbox {cm}^{-3}$	$11.1 \pm 2.0$	$\cdots$
*a*	au	$0.0177 \pm 0.0003$	$0.0883 \pm 0.0016$
*i*	deg.	$78.2 \pm 0.7$	$\cdots$
$T_\textrm{eq}$	K	$2038 \pm 39$	$912 \pm 18$
$S_\textrm{inc}$	$S_\oplus$	$2852 \pm 107$	$115 \pm 5$
$g_\textrm{surf}$	$g_\oplus$	$3.16 \pm 0.45$	$\cdots$

Parameter	Unit	Value	
Miscellaneous parameters			
$M_\star$	$M_\odot$	$0.959 \pm 0.049$	
$R_\star$	$R_\odot$	$0.979 \pm 0.025$	
$u_1$	$\cdots$	$0.42 \pm 0.04$	
$u_2$	$\cdots$	$0.24 \pm 0.03$	
$\gamma _\sigma$	$\cdots$	$1.24 \pm 0.04$	
$\langle f \rangle$	ppm	$-8.2 \pm 3.0$	
$\sigma _{K2}$	ppm	$70 \pm 1$	
$\gamma _\textrm{HARPS,RV}$	$\textrm{m}\,\textrm{s}^{-1}$	$0.703 \pm 0.002$	
$\sigma _\textrm{HARPS,RV}$	$\textrm{m}\,\textrm{s}^{-1}$	$1.36^{+0.54}_{-0.66}$	
$\gamma _\mathrm {HARPS-N,RV}$	$\textrm{m}\,\textrm{s}^{-1}$	$0.700 \pm 0.002$	
$\sigma _\mathrm {HARPS-N,RV}$	$\textrm{m}\,\textrm{s}^{-1}$	$0.44^{+0.53}_{-0.32}$	
$\gamma _\mathrm {HARPS,S-index}$	$\textrm{m}\,\textrm{s}^{-1}$	$0.175 \pm 0.030$	
$\sigma _\mathrm {HARPS,S-index}$	$\textrm{m}\,\textrm{s}^{-1}$	$0.93^{+1.14}_{-0.69}$	
$\gamma _\mathrm {HARPS-N,S-index}$	$\textrm{m}\,\textrm{s}^{-1}$	$0.174 \pm 0.030$	
$\sigma _\mathrm {HARPS-N,S-index}$	$\textrm{m}\,\textrm{s}^{-1}$	$1.28^{+1.33}_{-0.95}$	
$\lambda _\textrm{e}$	days	$206_{-51}^{71}$	
$\lambda _\textrm{p}$	$\cdots$	$7.5 _{ - 2.2 } ^ { + 1.8 }$	
$P_\textrm{GP}$	days	$37.6 _{ - 1.1 } ^ { + 0.7 }$	

$^a$ Minimum mass. BKJD refers to BJD–2454833.

### System stability and formation pathways

**Fig. 4:**
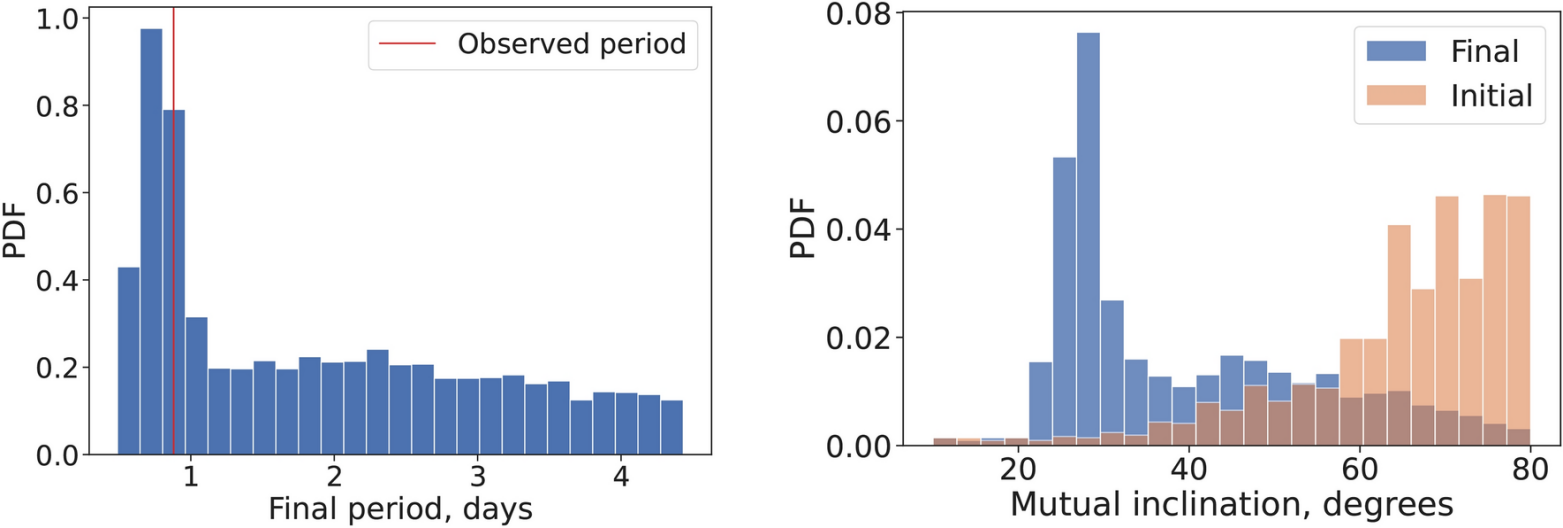
*Left panel*: Distribution of the final period of the inner planet, at the end of the secular simulations. The red line indicates the period of K2-360  b. *Right panel*: Distribution of mutual inclinations between the two planets, only for the realizations that end up with a USP (defined as any planet at less than one day orbit).

We applied the angular momentum deficit (AMD) stability criterion to set an upper limit on the mutual inclination ($i_\textrm{mut}$) between the inner and outer planets, following established methodologies^60–63^, resulting in $i_\textrm{mut} \lesssim 80^\circ$. Additionally, considering K2-360 b ’s current period of 0.88 days, we explored longer periods, hypothesizing that K2-360 b migrated to its current location after the protoplanetary disk dispersed. Understanding the initial orbit’s parameter space for K2-360 b is crucial for deciphering its migration mechanism. Many of the proposed scenarios for USP formation involve tidal dissipation of the inner planet, whether due to orbital eccentricity^64,65^ or planet obliquity^66^. If the migration is due to orbital eccentricity, the latter can be excited by dynamical interactions with the rest of the system. The migration can happen at either high ($e\sim 1$) eccentricity or low ($e\sim 0.3$) eccentricity, depending on the mechanism of eccentricity excitation^64,65,67^.

We explored the eccentric tidal migration scenario using secular simulations of two-planet gravitational interaction and tidal dissipation. We uniformly sampled initial periods of K2-360 b between 1 and 4.7 days, and with mutual inclinations between $10$ and $80^\circ$, for a total of via 4096 simulations, run over 6 Gyr (see Methods for details). Despite tidal parameter uncertainties, simulations revealed insights into the system’s possible initial configuration (Fig. 4). About 36% of the realizations concluded with K2-360 b orbiting in less than one day, and were particularly prevalent with large mutual inclinations. However, migration also occurred with low mutual inclinations, resulting in a peak distribution of 20–40 degrees for mutual inclinations after the migration occurs. The evolution of a two-planet system with high ($>40^\circ$) mutual inclination is due to the ZKL mechanism^68–72^. In this case, the perturbation from the outer planet excites the eccentricity of the inner, inducing rapid, high-eccentricity migration due to strong tides at the pericenter passage^64^. Such a significant initial mutual inclinations between the inner planets might be induced by mechanism such as stellar flybys, disk warping, or planet-planet scattering^73^. On the other hand, tidal migration can happen at low eccentricity^65,67^. Contrary to high-eccentricity migration, low-eccentricity migration requires long timescales and enough angular momentum deficit in the system to keep the inner planet at a small but significant eccentricity ($e \sim 0.2$) over long timescales (> 1 Gyr). The majority (> 90%) of the USPs formed in our simulations undergo high-eccentricity migration, favouring it over low-eccentricity migration, albeit the latter cannot be ruled out.

We also ran direct N-body simulations to explore the possibility that the planet experienced migration due to obliquity tides. While these simulations cannot be run until 6 Gyr due to computational reasons, they show that the orbital period of K2-360 b might undergo period decay due to obliquity tides (see Methods section). Another possibility is that the planet acquired its relative inclination after migrating inwards in a near coplanar configuration. Inward migration might be caused by disk torques during stellar episodic accretion events^74,75^, and the mutual inclination may be subsequently excited by stellar oblateness^76,77^.

### Interior structure

**Fig. 5:**
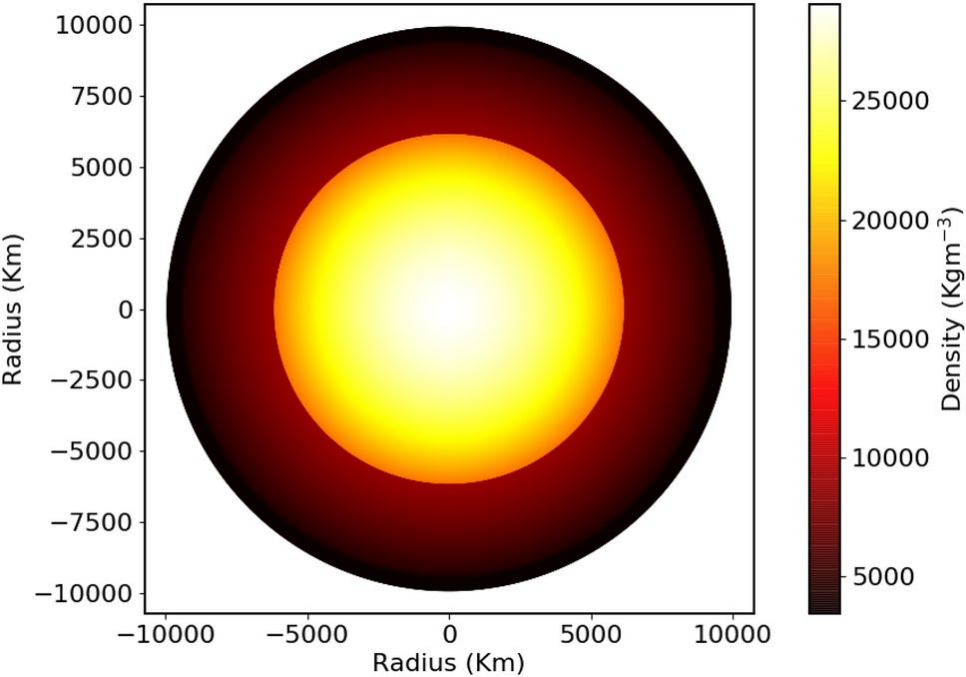
Visualization of the radial density variation from our interior composition model for K2-360 b.

**Fig. 6:**
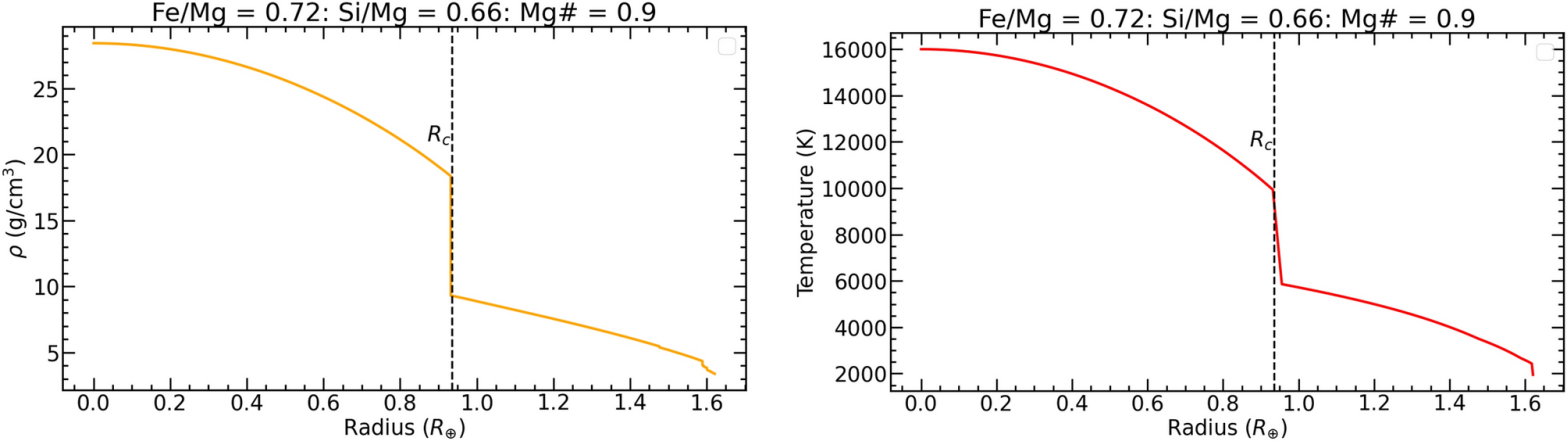
Density (left) and temperature (right) profiles of K2-360 b based on the observed chemical abundances of the host star.

We employed a terrestrial planet interior structure model to deduce the internal composition of K2-360 b^78^, assuming spherically symmetric differentiation into core, lower mantle, upper mantle, and crust shells. We utilized equations of state (EoS) specific to each layer’s composition to compute density and other thermoelastic properties^79–81^. By equating the planetary composition to the host star’s composition, elemental ratios (Fe/Mg, Si/Mg, Ca/Mg, Al/Mg) were constrained using spectroscopic data from K2-360^82–85^. With a Fe/Mg ratio of 0.72 and an Mg# (= Mg/[Mg + Fe]) of 0.9, an optimal core mass fraction (CMF) of 48 percent was determined, resulting in a radius of 1.6$R_{\oplus }$ and for a mass of 7.7$M_{\oplus }$, consistent with observational data. The density structure of our interior composition model is shown in Fig. 5**.** Notably, the CMF of 48 percent places K2-360 b closer to a Super-Earth than a Super-Mercury. An alternative model with a higher Fe concentration and a lower Si/Mg ratio yielded a CMF of 57 percent, resulting in a radius of 1.58$R_{\oplus }$ and a mass of 7.8$M_{\oplus }$. However, the Fe abundance implied by this model exceeds that observed in the host star. In theory an Fe/Mg value above 1.2, an Si/Mg of 0.5 and strong differentiation could move the CMF above 60 percent. It is plausible that being closer to the star, K2-360  b could have a Fe concentration high enough to make it a Super-Mercury. Given the data, however, the most likely CMF falls between 48 percent and 57 percent. The density and temperature profiles of K2-360 b are shown in Fig. 6. USP planets like K2-360  b have host star metallicity distributions similar to sub-Neptunes at longer periods, but unlike those of hot Jupiters^12^. If K2-360  b is the stripped core of a sub-Neptune, its current mass suggests an initial mass of 9–11 $M_{\oplus }$, assuming a 10–30% envelope fraction typical of sub-Neptunes^86^. With its high surface temperature, K2-360 b may possess a magma-rich surface^87,88^. An EoS that accurately models magma mantles would be able to give a better estimate of the planetary CMF.

## Discussion

**Fig. 7:**
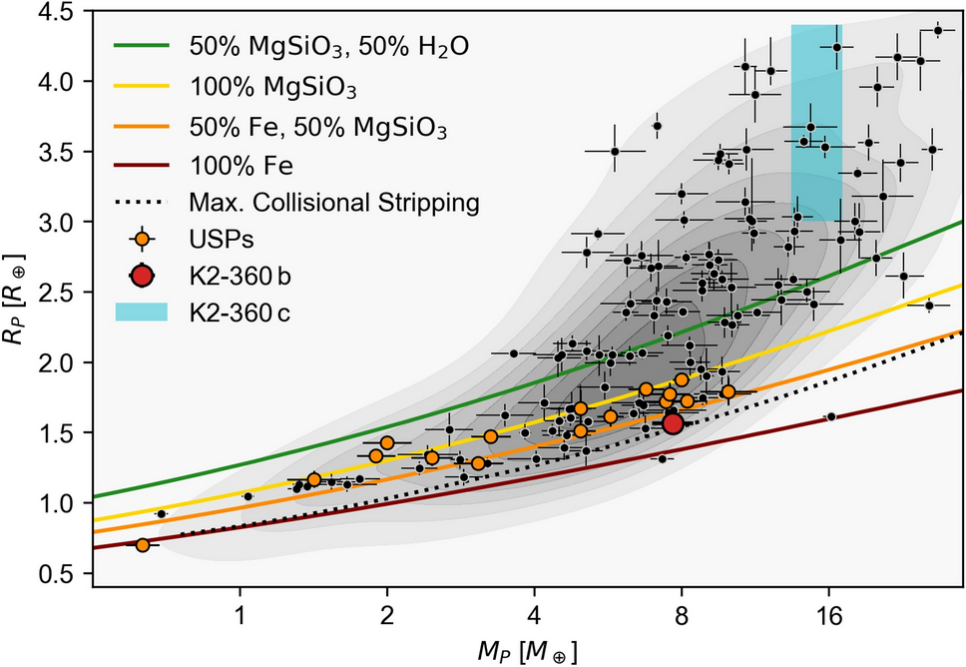
Mass-radius diagram showing the position of K2-360  b (red), as well as all known planets with masses and radii measured to better than 15% precision (black), with USP planets shown in orange. The known planet parameters are from the NASA Exoplanet Archive confirmed planets table, as queried on February 15, 2024, and a KDE of the population is shown in gray. The light blue shaded rectangle indicates the 1$\sigma$ range for the mass and radius of the non-transiting planet, K2-360  c, where the mass corresponds to the measured minimum mass (i.e. assuming $i\mathbf{=90}^\circ$) and the radius is estimated from a mass-radius relation^89^.

**Fig. 8:**
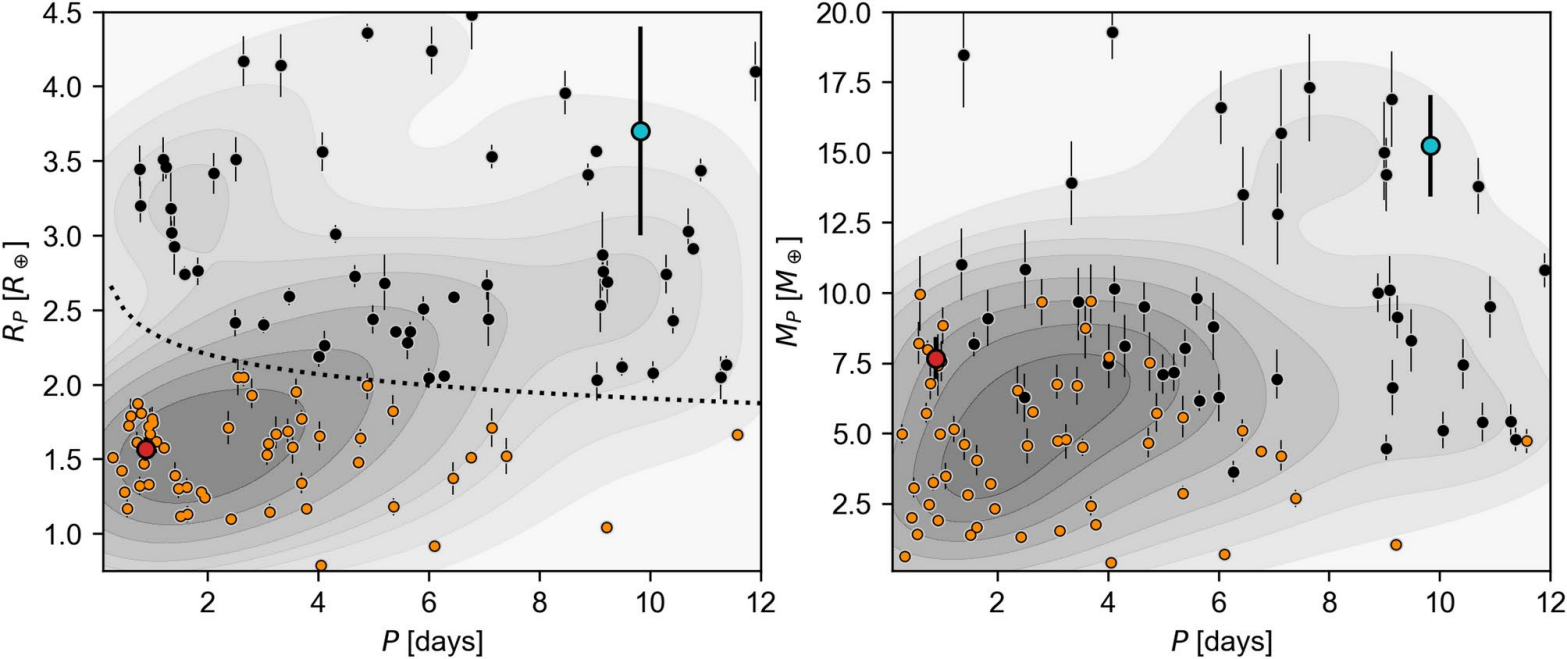
Period-radius (left) and period-mass (right) diagrams showing the positions of K2-360  b (red) and K2-360  c (blue), each showing the distribution of known planets, with a KDE of the distribution shown in gray. Note the radius of K2-360  c shown in the left panel was estimated from a mass-radius relation^89^ assuming $i=90^\circ$. As in Fig. 7, the known planet parameters were obtained from the NASA Exoplanet Archive, and only those planets with both radii and masses measured to better than 15% are considered. The dotted line shows the location of the radius valley^90^, and the planets below the radius valley are highlighted in orange in both panels. The KDEs were computed using only the data within the limits shown.

To place K2-360  b in context, in Fig. 7 we show its position in the mass-radius plane along with other precisely characterized planets. For comparison we also show theoretical composition models^91,92^; models including hydrogen envelopes are not shown, as the radii of such planets usually exceed 3$R_{\oplus }$ with as little as 0.1% $\hbox {H}_2$. The previously known planet parameters were obtained from the NASA Exoplanet Archive^93^ confirmed planets table on 2024 February 15, and only those planets with measurement precision better than 15% are displayed. We measure a precise (5.5$\sigma$) mean density of $11.1 \pm 2.0$ g $\hbox {cm}^{-3}$ for K2-360  b, which is consistent with a composition of 50% $\textrm{Fe}$ and 50% $\textrm{MgSiO}_3$. Other USPs are shown in orange, and tend to have similar densities greater than that of the Earth, consistent with rocky and iron-rich compositions. K2-360  b stands out as the most dense well-characterized USP planet, lying very close to the limit from collisional mantle stripping^94^ (dashed line in Fig. 7). The other extremely dense USP in the sample is GJ 367 b^95^ with a mean density of $10.2 \pm 1.3$ g $\hbox {cm}^{-3}$^96^, but we note that this is a super-Mercury with a 7.7 hour orbit around an M dwarf and is thus likely a fundamentally different kind of planet; indeed, GJ 367 b stands apart from the main population of USPs shown in Fig. 7. Since K2-360  c does not transit, we do not know its radius and can only measure a minimum mass, but we display the ± 1$\sigma$ credible region in blue; many of the previously known planets with similar masses have radii consistent with this estimate, i.e. comparable to that of Neptune. We also computed a kernel density estimate (KDE) of the known planet population (shown as gray contours), which peaks near the rocky super-Earth locus. We note that K2-360  b is more dense than all but one other USP, despite its location near the peak of the super-Earth distribution.

In Fig. 8 we show the positions of the K2-360 planets in both the period-radius and period-mass planes, compared to the population of well-characterized planets (precision $>15$% in both mass and radius). K2-360  b is clearly below the radius valley^90^, and both its radius and mass occupy densely populated regions of parameter space. On the other hand, K2-360  c appears to occupy a less well-populated region of the period-mass plane, although this could be a selection effect due to the increased difficulty of precise mass measurements at longer periods. We used an empirical mass-radius relation^89^ to predict the radius of K2-360  c given the measured minium mass, and obtained a radius of $3.6^{+0.7}_{-0.4}$ $R_{\oplus }$ (see left panel of Fig. 8). K2-360  c is thus likely to be Neptunian, unless its orbit is highly inclined, in which case it would be more massive and possibly larger. The datapoints nearest to K2-360  c in the period-mass plane (see right panel of Fig. 8) correspond to planets ranging in size from $\sim$3–5 $R_{\oplus }$. In particular, K2-360  c may be similar in nature to WASP-47 d^97^, TOI-220 b^98^, and TOI-1052 b^99^. Using its estimated radius from the mass-radius relation, we computed the maximum inclination of K2-360  c given its lack of transits in the *K2* photometry and compared this with the measured inclination of K2-360  b; we place a lower limit on their mutual inclination of $i_\textrm{mut} < 6.3^\circ$ (3$\sigma$), accounting for all uncertainties. Considering the upper limit from stability, we thus have $6^\circ \lesssim i_\textrm{mut} \lesssim 80^\circ$.

K2-360  b has an extremely high density, consistent with being a stripped planetary core with an iron-rich, rocky composition. Unlike some other dense USPs^100^, its internal structure appears to be more similar to Earth than Mercury. Any primary atmosphere the planet may once have had has likely been stripped by photo-evaporation. Highly irradiated close-in planets such as K2-360  b undergo thermal atmospheric escape^101^, which is largely driven by high energy (XUV) photons^102,103^. The timescale for low-mass planet photo-evaporation is determined by the relation between the planet radius and envelope mass for a given XUV flux, and this timescale is longest when the envelope mass fraction is of order a few percent, as increasing the envelope mass fraction quickly increases the planetary radius^104^. The age of the host star (likely >5 Gyr) implies a low present-day XUV flux and/or a fully evaporated primordial atmosphere, making it unlikely that H/He atmospheric escape could be detected by spectroscopic transit observations of K2-360  b; however, a secondary atmosphere may be detectable. To assess the viability of atmospheric studies of K2-360  b, we computed its transmission spectroscopy metric (TSM) and emission spectroscopy metric (ESM)^105^. While atmospheric characterization is possible for K2-360  b, the TSM ($8.9 \pm 4.2$) and ESM ($2.5 \pm 0.3$) values are relatively low, indicating that it is not likely to be among the most favorable targets for such studies with *JWST*; however, K2-360  b may be an interesting target for next generation large ground-based facilities^106^.

The formation scenario we examined is a high-eccentricity migration mechanism analogous to migration via secular chaos^107^. In our case, the high eccentricity is induced by the secular perturbations of an inclined companion, rather than by secular chaotic excitation in a multi-planet system. Similar to the secular chaos mechanism, our scenario produces a USP mutually inclined with respect to the outer companion. Another proposed mechanism for USP formation is the low-eccentricity migration due to secular resonances^108^. However, this mechanism requires smaller masses ( ${\lesssim }3$ $M_{\oplus }$) for the inner planet, which is in significant tension with the measured mass of K2-360  b. The low-eccentricity migration also requires a third close-in massive planet, which does not emerge from the RV data. Another caveat of the low-eccentricity migration is that requires the initial orbit to be a very short-period one (1–3 days) to begin with. Finally, obliquity tides have been proposed as a mechanism for USP migration^109^. This scenario requires an inclined, outer companion at a separation of ${<}10$ days, misaligned with the stellar spin, while the USP’s orbit is aligned with the stellar rotation axis. While we have no constraints on the stellar spin, the other requirements are consistent with our findings. On the other hand, high-eccentricity migration and obliquity migration are not mutually exclusive in principle. Obliquity tides might contribute to the USP migration, together with eccentricity tides induced by the ZKL mechanism.

Ever since radial velocity follow-up of CoRoT-7 b, a USP and the first known transiting super-Earth, revealed additional (non-transiting) planets in the system^110,111^, USPs have often been found in multiplanet systems. The possibility exists that even apparently single-planet USP systems^112^ have undetected outer planets due to a lack of RV data and/or the lower transit probability at longer periods; thus a single, dominant migration mechanism could potentially explain USPs. Nevertheless, distinct classes of USPs with differing formation mechanisms are also possible, e.g. coplanar (multi-transiting) systems^13,113,114^, and possibly non-coplanar, singly-transiting systems with outer planets detected only by RVs^8,95,96^. However, due to the extreme nature of a USP’s orbit, a non-transiting outer planet can be coplanar with a transiting USP. On the other hand, some multi-transiting systems containing a USP are not coplanar^9,115^. Consideration of differences in host star spectral types may also be important, although the these differences may be negligible across a wide range of FGKM stars^116^, becoming important only for mid or late M dwarfs such as TRAPPIST-1^117,118^. K2-360 falls into the latter category of singly-transiting, multiplanet USP systems, and due to the orbital inclination of the USP, we cannot rule out a present-day coplanar configuration; given the likelihood of migration via secular processes, however, a coplanar configuration is unlikely.

In conclusion, our intensive follow-up observations and analyses confirm and characterize the transiting USP planet K2-360  b. Our transit photometry unequivocally associates the signal with the target star K2-360, ruling out nearby sources. High-resolution imaging sets stringent upper limits on close stellar companions. Multi-year RV observations yield precise mass measurements for K2-360  b and reveal a non-transiting outer planet (K2-360  c) in a slightly eccentric orbit, with tight constraints on its minimum mass. High-resolution spectra provide enhanced characterization of the host star, complemented by detailed dynamical simulations and interior structure modeling. K2-360  b emerges as one of the densest known planets, notable even among the typically dense population of USPs. The minimum mass of K2-360  c suggests a likely Neptunian size and density, with our simulations indicating its potential role in inducing inward migration of K2-360  b via high-eccentricity tidal migration. Our findings suggest that systems like K2-360  b may represent a typical scenario for singly-transiting USP systems, where unseen outer companions are often missed due to lower geometric transit probabilities and RV semi-amplitudes.

## Methods

### Data collection

**Fig. 9:**
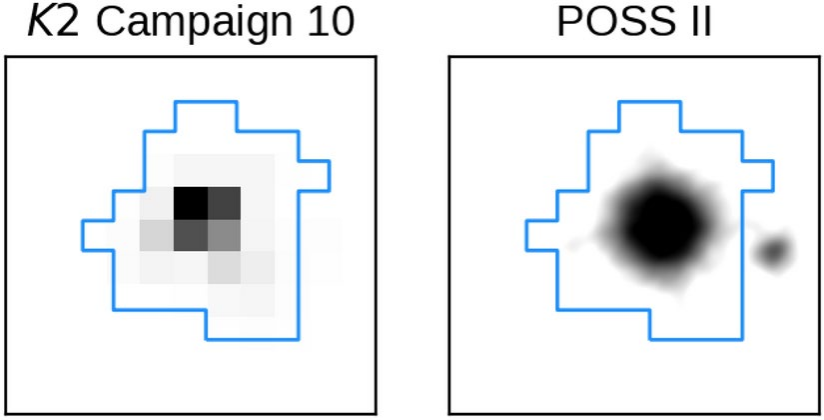
K2-360 11$\times$11 pixel *K2* “postage stamp” (left) and archival Palomar Observatory Sky Survey II (POSS II) image (right) centered on K2-360, with the EVEREST photometric aperture overplotted in blue. The nearby star EPIC 201595004 can be seen 13.6” away from K2-360 in the POSS II image, just outside the aperture.

*K2*. K2-360 was observed during Campaign 10 of the *K2* mission from 2016 July 6 to September 20, using channel 70 (module 20) of the *Kepler* photometer. A large pointing error during the first 6 days of the campaign resulted in low quality data, and soon after this was corrected the failure of module 4 powered off the *Kepler* photometer, resulting in a 14 day loss of data^119^. *K2* photometry suffers from correlated systematic noise induced by the roll of the *Kepler* spacecraft, which must be carefully modeled and subtracted to ensure robust parameter estimates. We used the EVEREST package^16,17^ to produce a corrected *K2* light curve. EVEREST selects an optimal photometric aperture and computes a systematics model based on pixel level decorrelation, a technique originally developed for the *Spitzer* mission^18^. The selected aperture is shown in Fig. 9, along with the *K2* pixel image from the middle of *K2* Campaign 10 and an archival image from the Palomar Observatory Sky Survey II. We recomputed the systematics model after masking out the transits^13^, thus ensuring that the transits were not partially fit out by EVEREST. The resulting light curve is dominated by stellar variability at the 1 ppt level, and is shown in the upper panel of Fig. 1.

*TESS*. K2-360 was observed by Camera 4, CCD 1 of the *TESS* photometer during Sector 46, in which $\sim$27 days of two-minute cadence data were taken from December 2-30, 2021. The observations were continuous except for a $\sim$2.5 day gap in the middle, when *TESS* science operations stop for data downlink near perigee. The *TESS* data were processed by the Science Processing Operations Center (SPOC) pipeline^120^ to produce a standard systematics-corrected light curve for transit searches called Presearch Data Conditioning Simple Aperture Photometry (PDCSAP)^121–123^, which are available for download from the Mikulski Archive for Space Telescopes (MAST)^124^. The PDCSAP light curve exhibit no visible spot modulation or other obvious features. We also checked the SAP light curve, which are not corrected for systematics, but sometimes retain a more accurate signature of spot modulation, which can be removed by the SPOC pipeline. The SAP light curve exhibits typical systematic trends near the beginning of each contiguous data segment, as well as a shallow, positively-sloped, approximately linear trend; the latter could possibly be due to spot modulation, and would suggest that the timescale of the signal is significantly longer than 27 days.

**Fig. 10:**
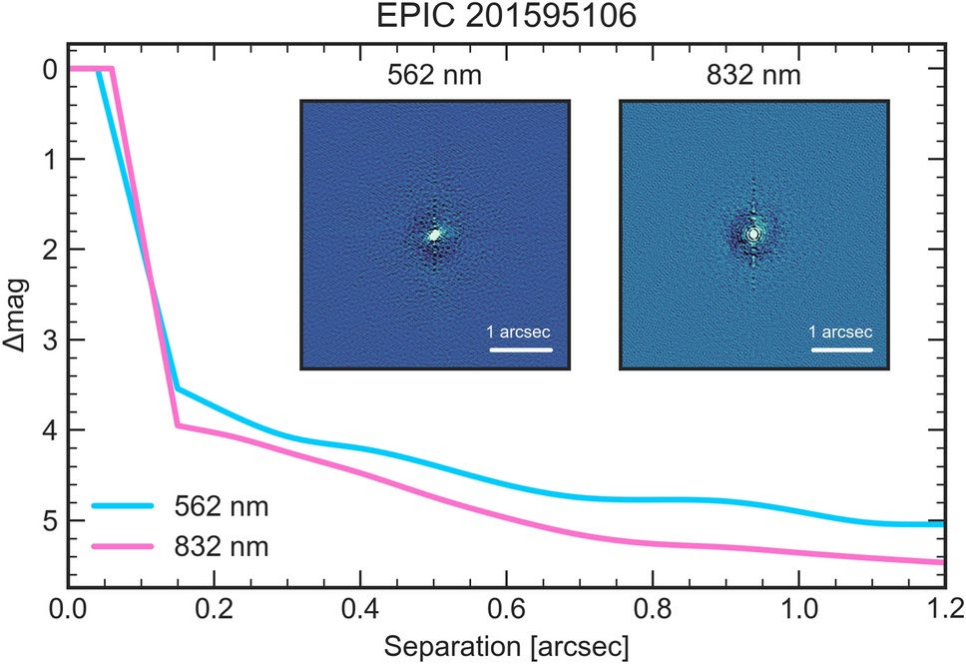
WIYN/NESSI reconstructed images and contrast curves. The inset images are 4.6”$\times$4.6”.

*NESSI*. On the night of UT 2017 March 18, we used the NASA Exoplanet Star and Speckle Imager (NESSI) instrument^22,23^ mounted on the 3.5 m WIYN telescope to observe K2-360 and nearby point-source calibrator stars, following standard practices^125^. The observations were conducted as part of NOAO observing program 2017A-0377 (P.I. Livingston). NESSI uses high-speed electron-multiplying CCDs to capture sequences of 40 ms exposures simultaneously in a “blue” band centered at 562 nm with a width of 44 nm, and a “red” band centered at 832 nm with a width of 40 nm. We produced reconstructed 256$\times$256 pixel images in each band (corresponding to 4.6”$\times$4.6”). No secondary sources were detected in the reconstructed images, and we used a series of concentric annuli centered on the target star to measure the background sensitivity. The reconstructed images and 5$\sigma$ contrast curves are shown in Fig. 10. This dataset was previously published in a catalog paper^13^, but we present it here for clarity.

**Fig. 11:**
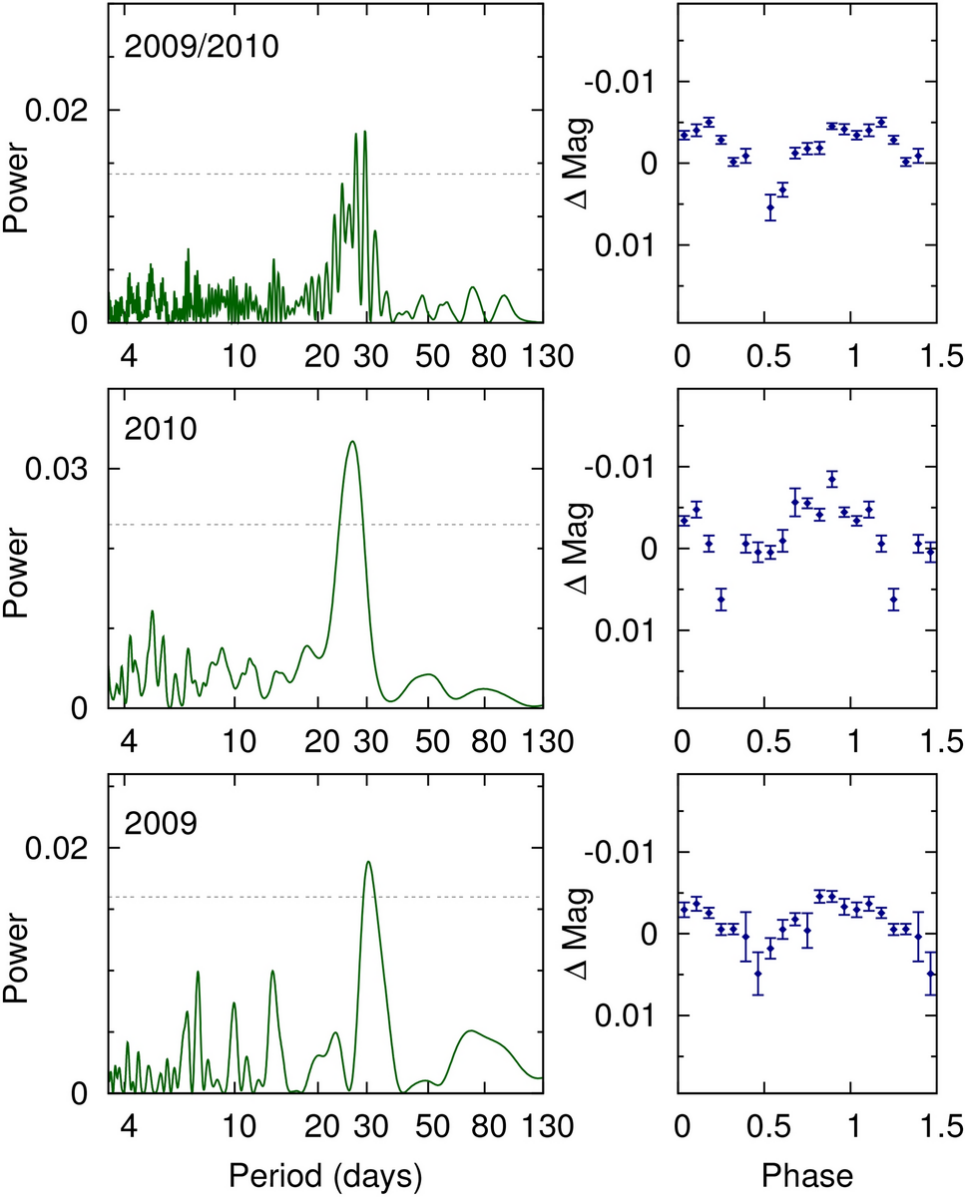
Lomb-Scargle periodograms of the WASP-South, Camera-225 photometry of K2-360, showing a periodicity of $28.0 \pm 1.4$ d. The top panel combines the data from the lower two panels. The horizontal line is the estimated 1% false-alarm probability. At right are folds of the data on the best period.

*WASP*. The field of K2-360 was observed by WASP-South during the WASP transit survey^31^ in 2009 and 2010. The WASP-South array of eight cameras, located in Sutherland, South Africa, used 200 mm, f/1.8 lenses with 400–700 nm filters backed by 2048 × 2048 CCDs, observing fields with a typical 10 min cadence. The observations reported here spanned 140 nights in 2009 (JD 245 4855 to 245 4997) and again in 2010 (JD 245 5220 to 245 5360). Later observations obtained with 85 mm lenses are less useful at the magnitude of K2-360. The precision of the photometry is insufficient to detect any transits of K2-360 b. Fig. 11 shows periodograms of the WASP photometry, as well as the binned photometry phased to the period correspondng to the highest peak in the power spectrum.

*MuSCAT*. On the night of 2018 February 23 UT we observed K2-360 with MuSCAT^21^, a simultaneous multi-band imager on the 1.88 m telescope in Okayama, Japan. The observing conditions were good, and we obtained time series photometry of a 6.1’$\times$6.1’ field centered on K2-360 in *g*, *r*, and *z* bands using exposures of 24, 15, and 43 seconds, respectively. The observations lasted from 2018 February 23 17:17 to 20:24 UT comprising a window $\sim$3 hours wide centered on the time of mid-transit predicted from the ephemeris^13^. We reduced the data and conducted differential photometry to produce a light curve of K2-360 following standard procedures^126^. We achieved photometric precision of $\sim$3 ppt, insufficient to detect the $\sim$0.2 ppt transit of K2-360 b, but the observations rule out the possibility that the signal arises from the nearby star EPIC 201595004 ($\rho = 13.62$”, $\Delta Kp = 5.839$ mag), which would have a depth of >5% accounting for dilution from K2-360. Thus, given the FPP of 0.2%^13^, the MuSCAT photometry enables validation of the planet at 99.8% confidence.

*HARPS and HARPS-N*. We carried out the spectroscopic follow-up of K2-360 with the High Accuracy Radial velocity Planet Searcher (HARPS) spectrograph^41^ mounted at the ESO-3.6 m telescope of La Silla Observatory, Chile. We acquired 66 high-resolution (R $\approx$ 115 000) spectra between 23 April 2017 and 13 May 2018 (UT), as part of the observing programs 099.C-0374, 0100.C-0808, and 0101.C-0829. Given the short orbital period of the transiting planet, we tailored our observing strategy to maximise the orbital phase coverage within each night, by acquiring at least two spectra in most of the observing nights. We reduced the data using the HARPS Data Reduction Software (DRS)^43^ and cross-correlate the spectra against a G2 numerical mask. We used the DRS to extract the full width at half maximum (FWHM), the bisector inverse slope (BIS), and the contrast of the cross-correlation function (CCF), and the Ca ii H  & K lines activity indicator ($\mathrm{log\,R}^{\prime }_\textrm{HK}$). Between 29 January 2017 and 10 March 2019 (UT), we also acquired 44 high-resolution (R $\approx$ 115 000) spectra with the HARPS-N spectrograph^42^ mounted at the 3.58 m Telescopio Nazionale Galileo (TNG) of Roque de los Muchachos Observatory (La Palma, Spain), via programs A34TAC_44, OPT18A_44, CAT18A_130, A37TAC_37, OPT18B_52, CAT18B_62, and A38TAC_26. We followed the same multi-visit strategy adopted with HARPS, i.e., we acquired at least 2 spectra per night in most of the observing nights. We reduced the spectra using the HARPS-N DRS and extracted the same activity indicators and line profile diagnostics as for HARPS. We also used SERVAL^44^ to compute additional activity indicators and line profile variation diagnostics, namely, the H$\alpha$, Na D lines indicators, the differential line width (dLW), and the chromatic index (CRX). SERVAL employs a template-matching algorithm specifically designed to derive precise radial velocities from high-resolution Echelle spectra of late K- and M-type dwarfs, but is also useful for earlier type stars like K2-360.

### Host star characterization

**Fig. 12:**
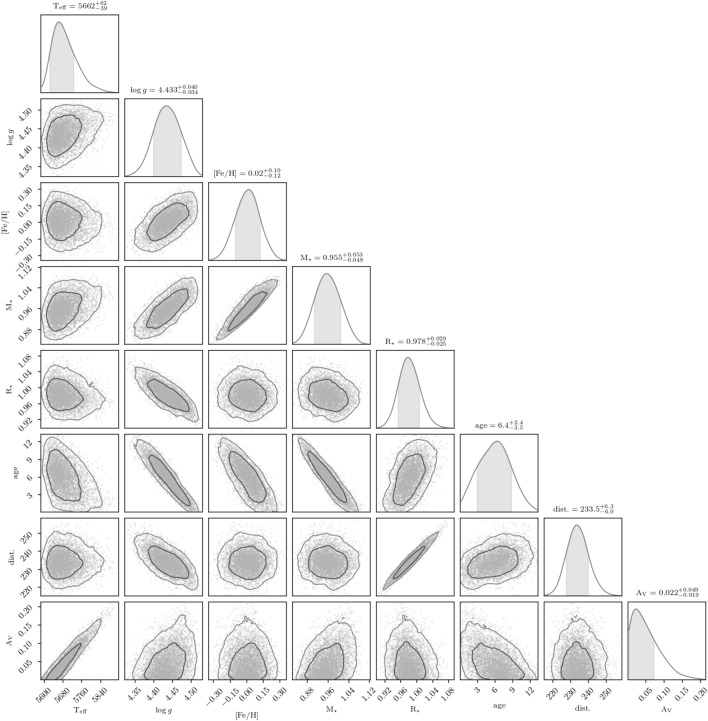
Joint posterior distribution of stellar parameters sampled with MultiNest via the isochrones interface to the MIST stellar evolution models. Note the gray shading of the marginal distributions show the 68% credible regions, and the values reported are centered on the mode.

We derived stellar parameters from coadded HARPS and HARPS-N spectra utilizing the SME analysis framework^24^. Macroscopic turbulence velocity was fixed at 3.1 $\textrm{km}\,\textrm{s}^{-1}$^25^, while microscopic turbulence velocities for HARPS and HARPS-N were set at 1.0 $\textrm{km}\,\textrm{s}^{-1}$ and 0.9 $\textrm{km}\,\textrm{s}^{-1}$, respectively^26^. From the HARPS data, we obtained $T_{\textrm{eff}}$ = 5550 ± 80 K, $\log g$ = 4.31 ± 0.14 (cgs), $[\text{ Ca }/\text{H}]$ = 0.00 ± 0.27 dex, $[\text{ Fe }/\text{H}]$ = − 0.10 ± 0.13 dex, and $v \sin i$ = 2.5 ± 1.5 $\textrm{km}\,\textrm{s}^{-1}$. Similarly, from the HARPS-N data, the derived parameters were $T_{\textrm{eff}}$ = 5512 ± 75 K, $\log g$ = 4.24 ± 0.11 (cgs), $[\text{ Ca }/\text{H}]$ = 0.03 ± 0.30 dex, $[\text{ Fe }/\text{H}]$ = − 0.09 ± 0.12 dex, and $v \sin i$ = 2.5 ± 1.5 $\textrm{km}\,\textrm{s}^{-1}$. We computed the weighted mean of these values, arriving at $T_{\textrm{eff}}$ = 5530 ± 55 K, $\log g$ = 4.267 ± 0.087 (cgs), $[\text{ Ca }/\text{H}]$ = 0.013 ± 0.201 dex, $[\text{ Fe }/\text{H}]$ = − 0.095 ± 0.088 dex, and $v \sin i$ = 2.5 ± 1.1 $\textrm{km}\,\textrm{s}^{-1}$. We computed a set of uniformly inferred stellar parameters using the isochrones package^27^ and the MIST stellar model grid^28^. The fit incorporated optical photometry and parallax from *Gaia* DR2^127,128^ (*G*,$B_\textrm{p}$, $R_\textrm{p}$, $\pi$), as well as NIR photometry from 2MASS^129^ (*J*,*H*,*Ks*) and *WISE*^130^ (*W*1,*W*2,*W*3). We added 0.1 mas of uncertainty in quadrature to the *Gaia* DR2 parallax uncertainty to account for systematics^131^. We sampled the posterior using MultiNest^132^, placing Gaussian priors on $T_{\textrm{eff}}$ and $[\text{ Fe }/\text{H}]$ based on the weighted mean SME results from the coadded HARPS and HARPS-N spectra, which resulted in the following parameter estimates: $T_{\textrm{eff}}$  = $5679^{+60}_{-39}$ K, $\log g$  = $4.435 \pm 0.035$ (cgs), $[\text{ Fe }/\text{H}]$  = $0.01 \pm 0.10$ dex, $M_{\star }$  = $0.958 \pm 0.048$ $M_{\odot }$, $R_{\star }$  = $0.980 \pm 0.025$ $R_{\odot }$, age = $6.0^{+2.6}_{-2.9}$ Gyr, distance = $233.81 \pm 5.84$ pc, and $\mathrm {A_V}$=$0.04^{+0.05}_{-0.03}$ mag. We also used catalogue photometry and the *Gaia* distance to infer a set of stellar parameters using the astroARIADNE package^29^, which employs a Bayesian model averaging scheme over a number of fits to different stellar atmospheric model grids. Following other applications of this method^133^, we find $T_{\textrm{eff}}$  = $5640^{+29}_{-41}$ K, $\log g$  = $4.247 \pm 0.064$, $[\text{ Fe }/\text{H}]$  = $-0.0817^{+0.055}_{-0.063}$, $M_{\star }$  = $0.914^{+0.044}_{-0.050}$ $M_{\odot }$, and $R_{\star }$  = ${0.995}^{+0.025}_{-0.021}$ $R_{\odot }$. While there is mild tension between some of these values and the respective results from MIST above, the agreement is within $\sim$1$\sigma$, suggesting the inferred stellar parameters are reasonably robust even when marginalized over a number of different models. We adopt the MIST results for the stellar parameters used in this work, which are also presented in Table 2. The posterior distributions from the **isochrones** fit are shown in Fig. 12.

**Table 2:** Identifiers, coordinates, proper motion, parallax, photometry, and fundamental parameters of K2-360.

Parameter	Value	Source
Main identifiers		
EPIC[SPAN]201595106		EPIC
2MASS[SPAN]J12145289+0158057		ExoFOP
*Gaia* DR23701123406596146048		*Gaia* DR2
Equatorial coordinates, parallax, and proper motion		
R.A. (J2015.5)	$12^\textrm{h}14^\textrm{m}52.8872^\textrm{s}$	*Gaia* DR2
Dec. (J2015.5)	$+00^\textrm{d}07^\textrm{m}52.3605^\textrm{s}$	*Gaia* DR2
$\pi$ (mas)	$4.2803 \pm 0.0493$	*Gaia* DR2
$\mu _\alpha$ (mas $\hbox {yr}^{-1}$)	$6.097 \pm 0.092$	*Gaia* DR2
$\mu _\delta$ (mas $\hbox {yr}^{-1}$)	$-24.247 \pm 0.054$	*Gaia* DR2
Optical and near-infrared photometry		
*Kp*	11.678	EPIC
*G*	$11.6477 \pm 0.0002$	*Gaia* DR2
$B_\textrm{p}$	$12.0012 \pm 0.0011$	*Gaia* DR2
$R_\textrm{p}$	$11.1512 \pm 0.0010$	*Gaia* DR2
*J*	$10.588 \pm 0.019$	2MASS
*H*	$10.262 \pm 0.023$	2MASS
*Ks*	$10.218 \pm 0.021$	2MASS
*W*1	$10.158 \pm 0.023$	All*WISE*
*W*2	$10.218 \pm 0.021$	All*WISE*
*W*3	$10.086 \pm 0.072$	All*WISE*
Fundamental parameters		
$L_\star$ ($L_\odot$)	$0.893 \pm 0.015$	*Gaia* DR2
$v \sin i$ ($\textrm{km}\,\textrm{s}^{-1}$)	$2.5 \pm 1.1$	This work
$P_\textrm{rot}$ (d)	$28.0 \pm 1.4$	This work
$T_{\textrm{eff}}$ (K)	$5679^{+60}_{-39}$	This work
$\log g$ (cgs)	$4.435 \pm 0.035$	This work
$[\text{ Fe }/\text{H}]$ (dex)	$0.01 \pm 0.10$	This work
$M_{\star }$ ($M_{\odot }$)	$0.958 \pm 0.048$	This work
$R_{\star }$ ($R_{\odot }$)	$0.980 \pm 0.025$	This work
Age (Gyr)	$6.0^{+2.6}_{-2.9}$	This work
Distance (pc)	$233.81 \pm 5.84$	This work
$\mathrm {A_V}$ (mag)	$0.04^{+0.05}_{-0.03}$	This work

### Photometric time series analysis

#### Transit search

**Fig. 13:**
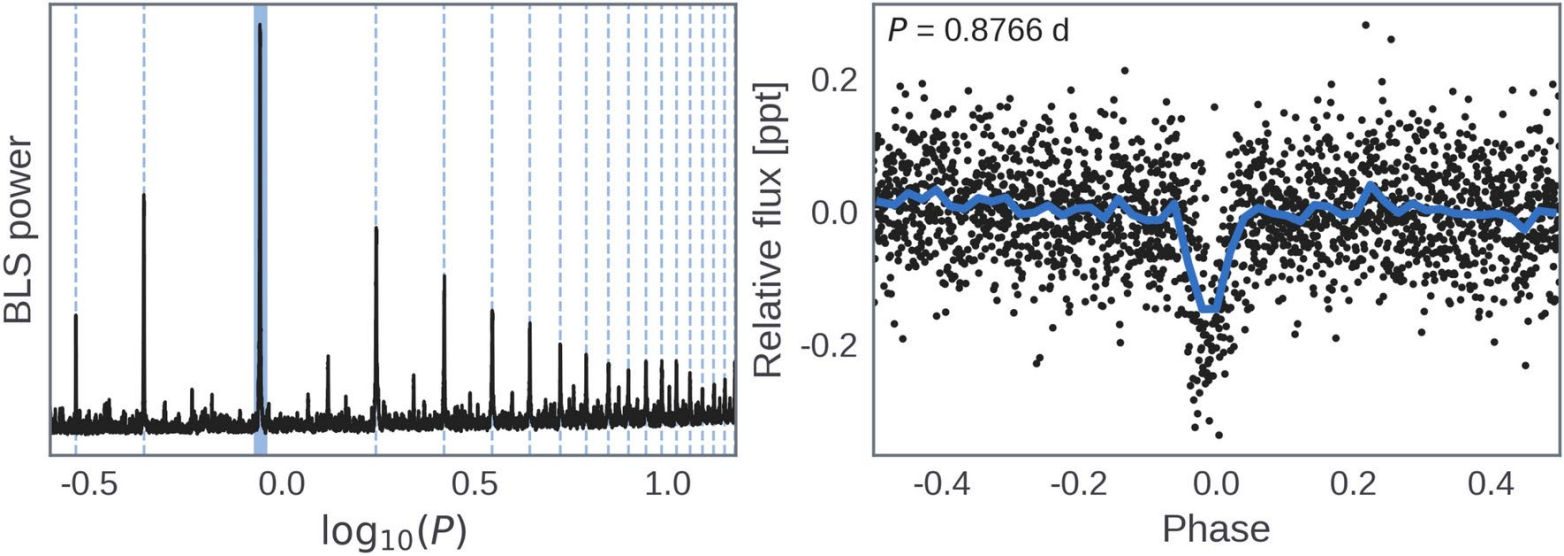
Left: BLS periodogram showing the orbital period of the transiting planet (shaded blue) and its harmonics (dashed blue lines). Right: *K2* photometry folded on the peak BLS period (black), and binned by a factor of 50 (blue).

We recovered the previously reported USP candidate^13^ from the *K2* light curve using the Box-Least-Squares (BLS) algorithm^19^, after modeling and subtracting stellar variability with a GP model^20^. We searched for additional transiting planets by re-running BLS on the *K2* light curve after masking out the transits of the USP, but no further signals were found. The BLS periodogram and phase-folded USP signal are shown in Fig. 13. We also searched the *TESS* Sector 46 PDCSAP light curve of K2-360 for transits, but the precision was too low (2 ppt RMS) to detect the shallow (0.2 ppt) transits of K2-360 b.

#### Stellar rotation

We searched the *K2* light curve for evidence of rotation-induced stellar spot modulation by computing the Lomb-Scargle periodogram and auto-correlation function (ACF) of *K2* photometry, but the results were inconclusive, perhaps due to the large data gap leading to only $\sim$47 days of useful data. The uncertainty from the *K2* photometery is large enough that the data is not very useful on its own, but taken together support a value for $P_\textrm{rot}$ in the range of $\sim$20–40 days, which is consistent with some of the peaks seen in the spectroscopic acitivity indicators and the expectation from $v \sin i$. The field of K2-360 was observed by WASP-South during the WASP transit survey^31^ in 2009 and 2010 using an array of eight cameras equipped with 200 mm lenses, but the photometric precision was insufficient to detect any transits of K2-360 b. A standard analysis of the WASP datasets^32^ revealed a significant periodicity with an amplitude of  3 mmags and false-alarm probability (FAP) lower than 1%. The periodogram of the combined dataset showed significant power in the 26–30 day range, which is consistent with the $v \sin i$ within the uncertainties. The period was found to be 30 ± 2 days and 26 ± 2 days in the 2009 and 2010 data, respectively. The periodograms and folded photometry are shown in the lower panels of Fig. 11. We note that while the estimates are consistent with each other, photometric rotational modulation is unlikely to be coherent over the timescale of the two datasets. With a periodicity near the lunar cycle we investigated the possibility of moonlight contamination (either from direct illumination of the lenses or by affecting the sky background). This has been seen in some WASP-South data, though usually in data from the 85 mm lenses, which are more affected by both issues than the 200 mm lenses. We thus examined the periodograms of four nearby stars of similar brightness in the same field, processed in the same way. None of these exhibit the periodicity that we see for K2-360, suggesting that it is not caused by moonlight contamination. The $v \sin i$ of 2.5 $\textrm{km}\,\textrm{s}^{-1}$ measured from the coadded HARPS and HARPS-N spectra corresponds to a maximum rotation period of $\sim$30 days (assuming $i_\star = 90^\circ$), and the 1$\sigma$ lower bound of 1 $\textrm{km}\,\textrm{s}^{-1}$ corresponds to $\sim$50 days. However, this is close to the instrument resolution and about the amplitude at which other complex broadening effects become significant; as such it may be more realistic to interpret this value as an upper limit. Further periodogram analyses of spectroscopic activity indicators also revealed peaks at $\sim$15 and $\sim$28 days. The rotation period is thus likely to be $\sim$28 days, with a starspot configuration resulting in significant power at the first harmonic. For final estimate of $P_\textrm{rot}$ we compute the weighted mean of the two WASP constrainted, resulting in $28.0 \pm 1.4$ d.

### Light curve modeling

We performed a stellar variability fit to the *K2* data using exoplanet^35^ and PyMC3^36^. We modeled the stellar variability as a GP with a simple harmonic oscillator (SHO) kernel, which represents a stochastically-driven, damped harmonic oscillator and has the following power spectral density:

$$\begin{aligned} S(\omega ) = \sqrt{\frac{2}{\pi }} \frac{S_0\,\omega _0^4}{(\omega ^2-{\omega _0}^2)^2 + {\omega _0}^2\,\omega ^2/Q^2} \end{aligned}$$

The hyperparameters $S_0$, $\omega _0$, and *Q* were explored in log space, with wide (uninformative) Gaussian priors. To avoid overfitting, we included a jitter term to account for possibly underestimated photometric uncertainties. Before fitting the GP we masked out the transits using twice the transit duration, as determined by the results of our BLS search. We found *maximum a posteriori* (MAP) parameter estimates using the gradient-based BFGS algorithm^37^ implemented in scipy.optimize. The light curve and best-fit GP model are shown in Fig. 1, along with the residuals and histogram comparing the residuals to the total noise determined by the fit. We then performed a transit fit to the *K2* data after removing the GP variability model computed above, again relying on exoplanet and PyMC3 for inference, as well as Starry^38^ for the transit model. We included a bias term $\langle f \rangle$, a white noise scale factor $\gamma _\sigma$, and assumed a quadratic limb darkening law under a transformation ($u_1$, $u_2$) for efficient sampling^134^. We used uninformative priors for the transit parameters ($T_0$, *P*, $R_P/R_\star$, *b*), and Gaussian priors on the stellar mass, radius, and limb darkening parameters, based on our results in Table 2. We used an initial MAP estimate to identify and remove 5$\sigma$ outliers, and then re-fit the model. We then initialized Hamiltonian Monte Carlo (HMC) sampling of the posterior via the “no U-turn sampling” (NUTS) algorithm^39^ implemented in PyMC3. The Gelman-Rubin statistic^40^ was <1.001 and the sampling error was $\lesssim$1% for all parameters, which indicated that the sampler was well-mixed and that a sufficient number of independent samples had been obtained. The resulting parameter estimates are in Table 1, along with the total white noise accounting for the scale factor ($\sigma _{K2}$). Fig. 2 shows the phase-folded (and detrended) photometry with the best-fitting transit model.

### Spectroscopic time series analysis

#### Frequency analysis

To accurately measure a planetary mass from RVs, it is necessary to search for potentially confounding signals from stellar activity, as well as additional massive bodies in the system. We thus conducted frequency analyses of the RVs and a range of stellar activity indicators from the HARPS and HARPS-N spectra using two different pipelines, DRS^43^ and SERVAL^135^. To account for instrumental offsets between HARPS and HARPS-N, we tested different methods to combine the time series measurements from each instrument into a single dataset for frequency analysis. In the first method (1) we used the mean of all measurements taken on BJD 2458220, 2458221, 2458222, and 2458223 to estimate the offsets, as near-simultaneous data were obtained with both instruments at these epochs. We also tested a variant (2) of this method that computed the median difference between measurements taken within 2.4 hours of each other. Finally, we tested a simple method (3) using the median offset computed from the full time series from each instrument. We found that method 3 performed significantly better than method 1, and about as well as method 2, as determined by visual inspection of the Lomb-Scargle periodograms of each instrument’s time series and the combined time series; we speculate that this behavior could be explained by the statistical power of the median over the full time series as opposed to smaller sub-samples. We opted to conduct frequency analyses on the combined time series produced via method 3, due to its simplicity and performance.

**Fig. 14:**
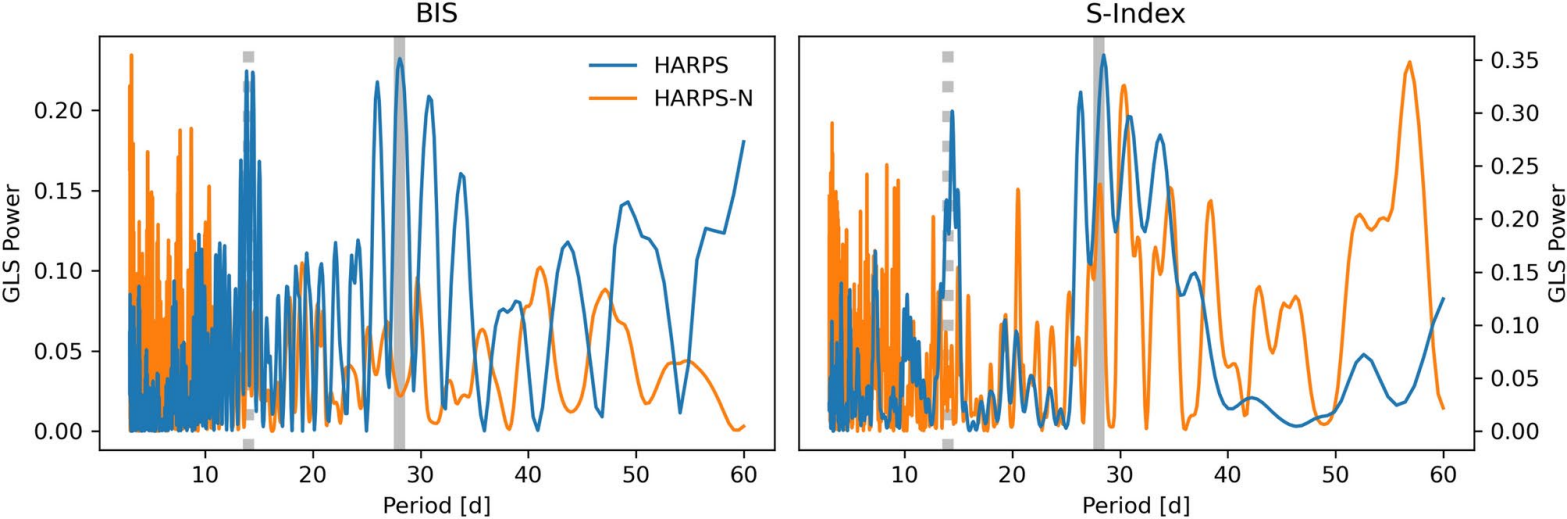
GLS periodograms of the BIS (left) and S-Index (right) activity indicator time series. The vertical gray and gray-dashed lines denote the rotation period and its first harmonic, respectively.

We computed GLS periodograms of the combined HARPS and HARPS-N RV measurements and activity indicators from the DRS and SERVAL pipelines, as well as the window function of the observations. As when determining the offset method used to combine time series from both instruments, we computed the periodograms for HARPS and HARPS-N separately, as well as for the combined dataset. We first visually inspected the periodograms computed from the DRS time series, and found the HARPS-N dataset to be noisier than the HARPS dataset, with fewer high SNR peaks and more aliases. In Fig. 14 we show the GLS periodograms of the BIS and S-index activity indicators, which show clear peaks near the stellar rotation period and its first harmonic in the HARPS data.

**Fig. 15:**
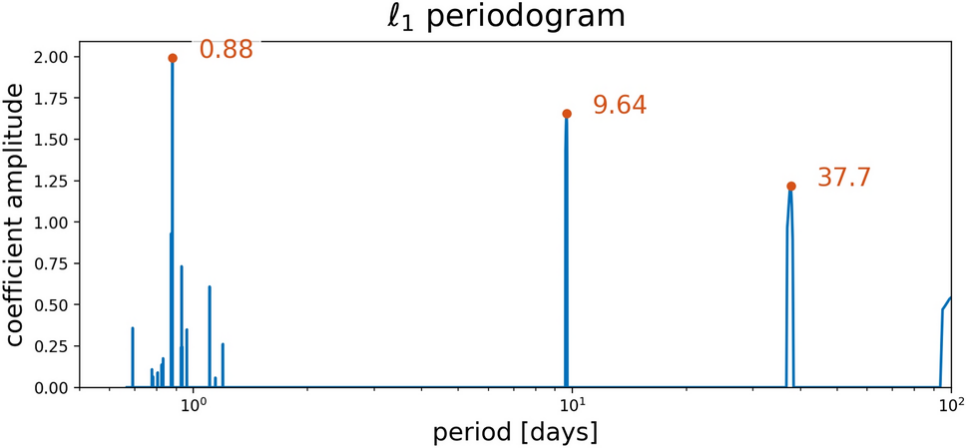
$\ell _1$ periodogram of the RVs.

**Fig. 16:**
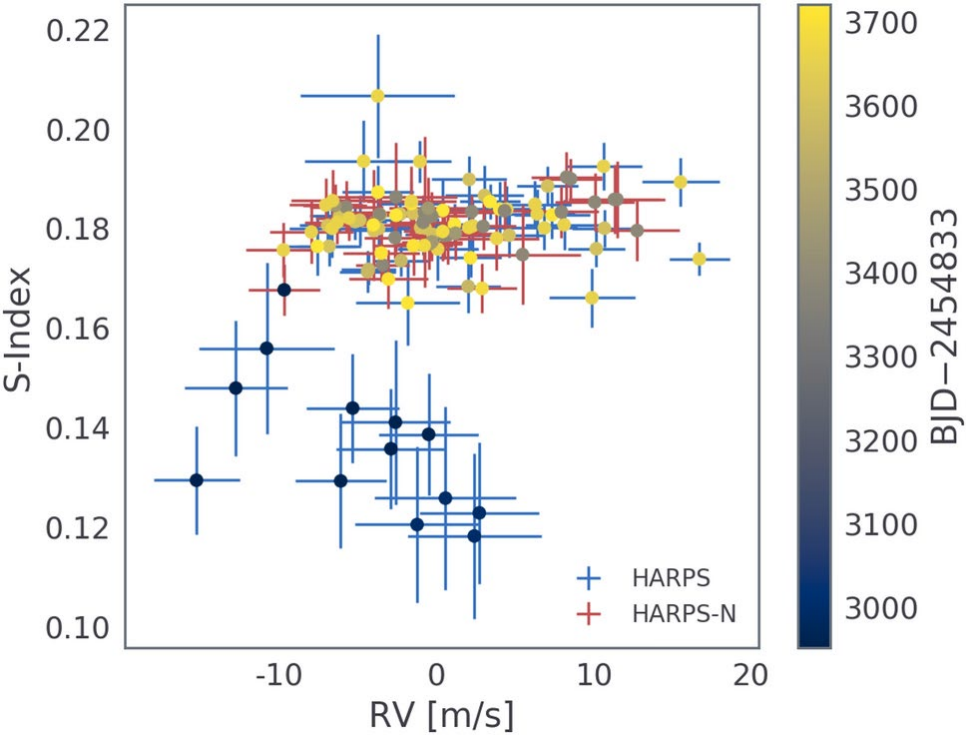
S-Index vs RV, with the color of the error bars corresponding to the instrument, and the color of the data point corresponding to the time of observation. The data from 2017 exhibit distinctly lower S-Index values than the subsequent observing seasons, possibly due to magnetic activity cycles^138^.

We also computed $\ell _1$ periodograms^46,136^ of the RVs and S-Index (see Fig. 15) using a publicly available code^47^. The optimization underlying the $\ell _1$ periodogram can more effectively recover sparsely- or irregularly-sampled signals in the presence of noise, and follows from the theory of compressed sensing^137^. Sometimes called the ‘taxicab’ distance, the $\ell _1$ objective function is the sum of absolute differences, as opposed to the Euclidean distance ($\ell _2$). The $\ell _1$ periodogram has the advantage of inducing sparsity in the representation of the data, which is a desirable characteristic when only a small number of significant signals are expected to be present. In the case of a star with orbiting planets, only a few signals should be present (one per planet and the star). We assumed a jitter value of 1 $\textrm{m}\,\textrm{s}^{-1}$ added in quadrature to the nominal RV uncertainties, a 0.5 $\textrm{m}\,\textrm{s}^{-1}$ calibration error, and a simple red noise model consisting of an exponential kernel with amplitude 1 $\textrm{m}\,\textrm{s}^{-1}$ and a timescale of 10 days. Fig. 15 shows the resulting $\ell _1$ periodogram, which exhibits a clear peak at the orbital period of the USP, as well as a nearly equal amplitude peak at $\sim$9.6 days, which we interpret as a second planet due to the absence of any counterpart in the activity indicators. There is also a significant signal at 37.7 days with a slightly lower amplitude, which we interpret as arising from stellar activity. We conclude that RV activity with a similar period to the stellar rotation period is likely caused by stellar activity, but that this signal is incoherent due to the evolution of the active regions over longer timescales, as can be seen in the behavior of the S-index shown in Fig. 16.

**Fig. 17:**
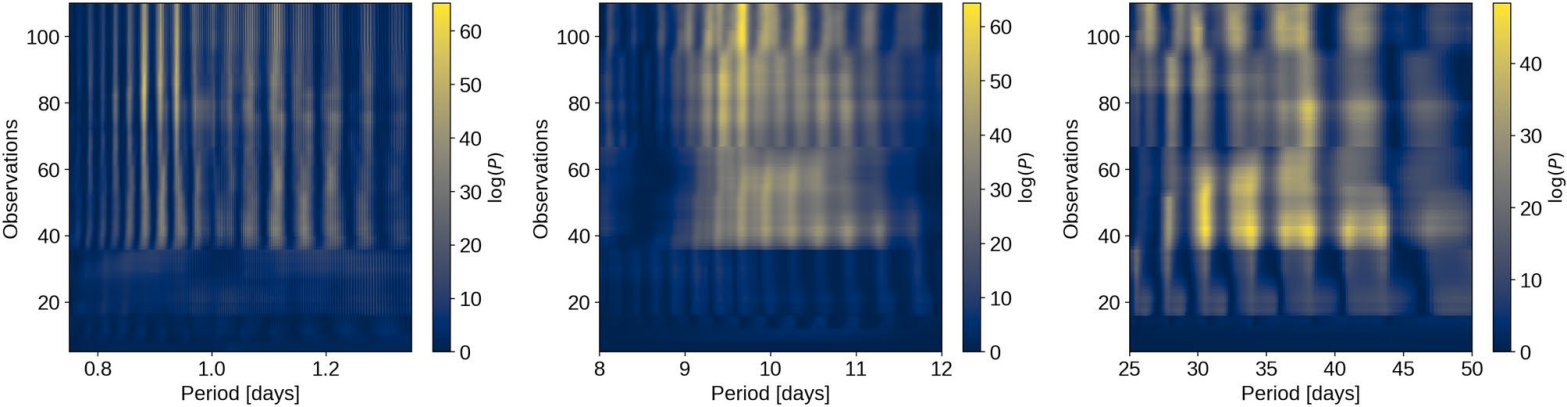
Stacked Bayesian Generalized Lomb Scargle periodograms near the periods of the USP planet (left), the non-transiting planet (middle), and the stellar activity signal (right).

The planetary Doppler signals are coherent over time, so they can be seen to get stronger as a function of the number of measurements. To examine this effect in the RV data, we computed the Stacked Bayesian GLS periodogram^49^ centered on the USP, the 9.8 day, and the longer period signals (see Fig. 17). The USP and 9.8 day signals can be seen to increase in strength with the addition of data, as expected for coherent signals, whereas the signals near the stellar rotation period do not. This further reinforces the interpretation of the USP and 9.8 day RV signals as planetary, and the longer period signals as stellar in origin.

#### FCO analysis

**Fig. 18:**
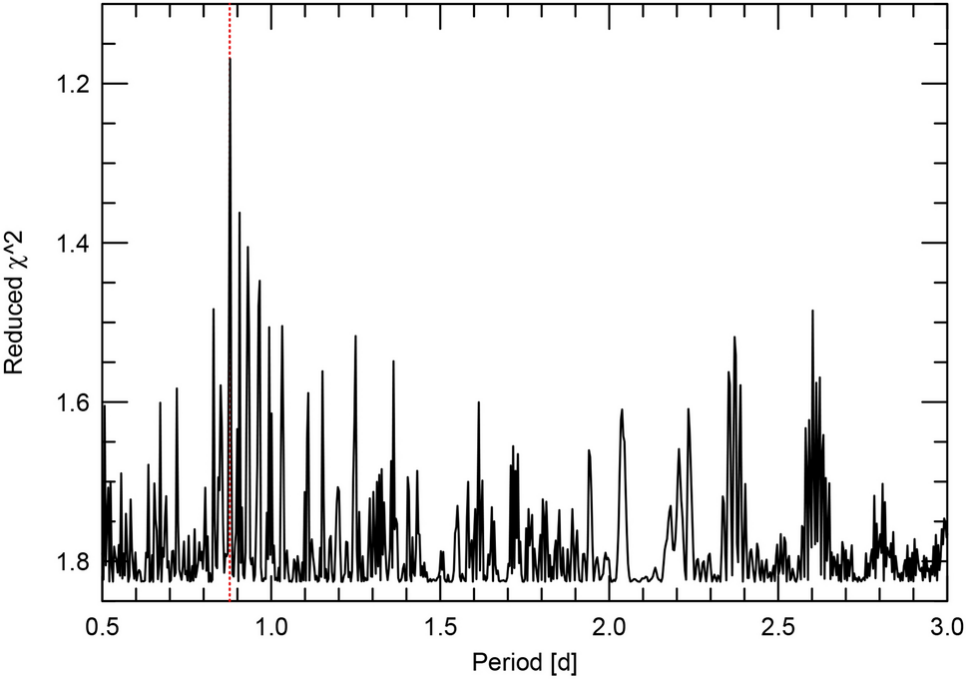
The FCO periodogram of the RV data. The reduced $\chi ^2$ is minimized for the transit period (dashed vertical line).

**Fig. 19:**
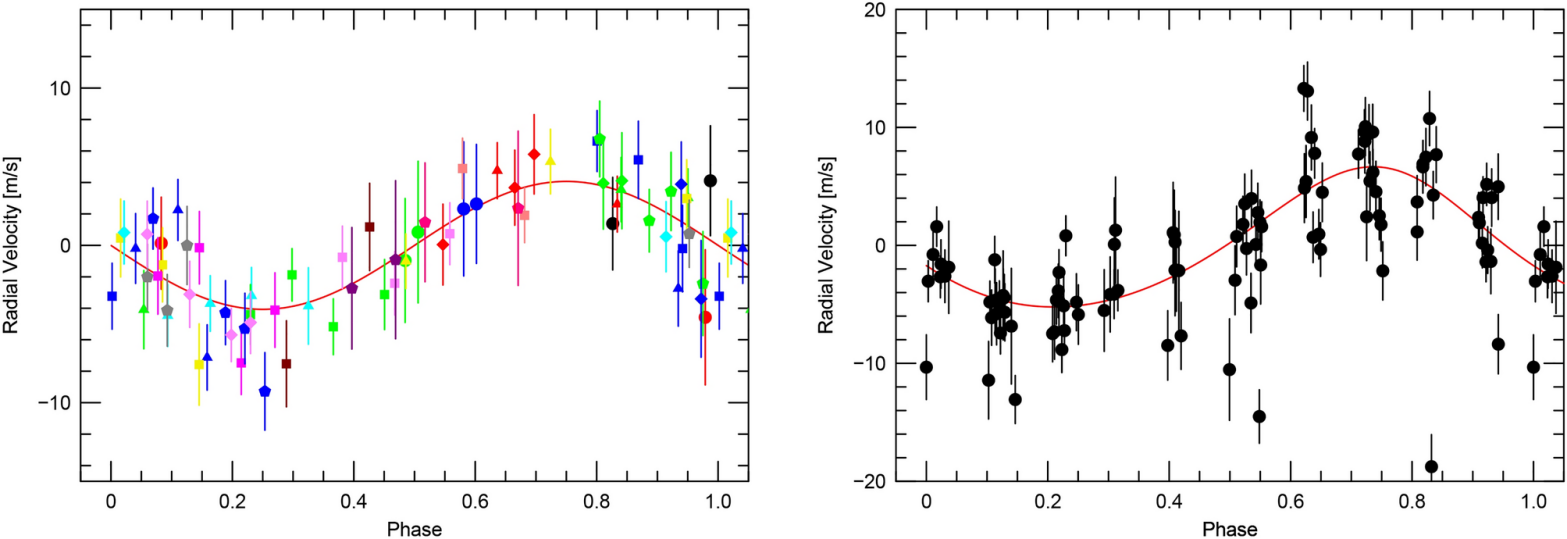
The phase-folded RV curve of K2-360 b from the FCO method; RVs for individual time chunks are shown with the same symbol and color (left). The orbital fit to the 10 d RV variations (right).

**Fig. 20:**
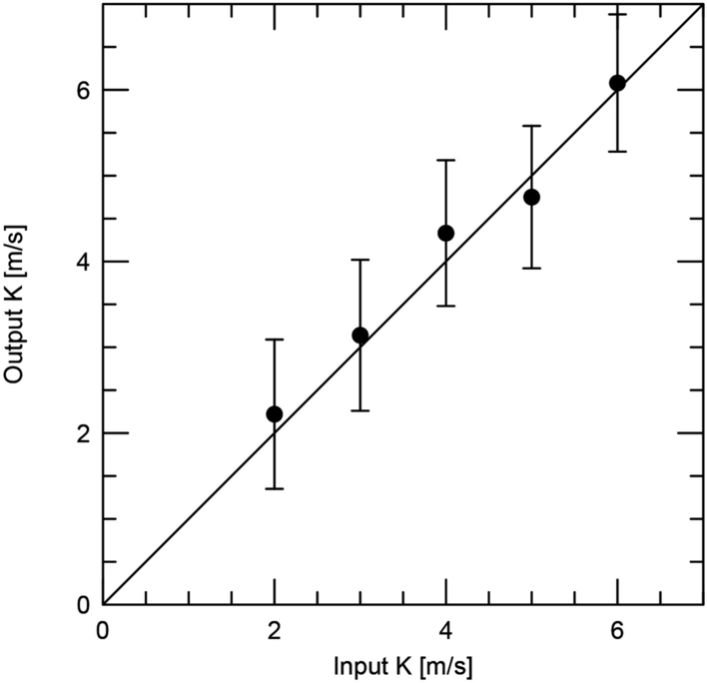
Simulations of the output *K*-amplitude derived using the FCO method as a function of the input amplitude. The line indicates a slope of unity.

The RV measurements have up to four measurements taken in one night, which is about 20 % of the orbital curve of the transiting planet. This data set is thus ideal for applying the so-called “floating chunk offset” (FCO) method^50^. To use FCO one divides the data into time chunks consisting of two or more measurements spanning several hours that were taken in one night. It assumes that the RV variations during that time come predominantly from the orbital motion of the short period planet and other variations such as those due to stellar rotation or long period planets are constant during the night. One then fits an orbit to the data keeping the period and phase fixed to the transit values, but allowing the zero point offset in each time chunk to float. The method is also a way of naturally combining different data sets that have their own zero point offsets as well as accounting for systematic errors, so long as these have variations that are longer than the length of the time chunks.

The FCO method can be used as a periodogram by fitting the data using different trial periods and then examining the reduced $\chi ^2$. This is shown in the lower panel of Fig. 18 for the period range 0.5–3 d. The y-axis is plotted with decreasing $\chi ^2$ upwards so that one can make a more direct comparison to standard periodograms where a peak signifies a signal. The reduced $\chi ^2$ is clearly minimized at the transit period. We would suspect a signal at this period even without the transit information.

The left panel of Fig. 19 shows the resulting RV curve after applying the FCO method and removing the calculated zero points. Points with the same color and symbol indicate RV measurements from an individual chunk so that one can assess how well the RV measurements in a single night follow the orbital curve. The resulting *K*-amplitude is 4.54 ± 0.88 m $\hbox {s}^{-1}$.

There is a concern that since FCO can vary the zero point in each chunk that it may be introducing a signal in the data at the transit period. We tested this by creating simulated data sets with periodic signals that were sampled in the same way as the observations.

A periodogram analysis after removing the RV variations of the transiting planet shows that a significant signal at 10 d with an amplitude of 5.9 m $\hbox {s}^{-1}$ (see below). In the raw periodogram there are low frequency signals (*P* > 17 d), but these disappear when one removes the 0.87 d and 10 d signals. Even if present, these would produce little RV variations in a few hours, the time span of the chunks. The orbital variations of the the USP planet were then inserted and random noise with a $\sigma$ = 2.3 m $\hbox {s}^{-1}$ was added. This value represents the median error of the RV measurements. We then divided the chunks in the same way as the previous analysis. For good measure we introduced a large (several km $\hbox {s}^{-1}$) offset in each time chunk to test the robustness of the results.

Figure 20 shows the fitted output *K*-amplitude as a function of the input value over the range *K* = 2–6 m $\hbox {s}^{-1}$, FCO is able to recover the input amplitude over this range so we can be confident that the method is not introducing a systematic error into our determination of the *K*-amplitude.

It is possible to fit eccentric orbits using the FCO method. Doing so results in a slight eccentricity (*e* = 0.04) but with a large error (0.13). There is no strong evidence for any eccentricity, so this was fixed to zero in the final solution. We also allowed the transit ephemeris to vary in fitting the orbit. The FCO result differed by only 0.01 d from the value derived from the transits. In other words, the orbital solution using the RV data alone is able to reproduce the transit period and ephemeris. The final orbital parameters are listed in Table 3 where we have used the photometric ephemeris.

The GLS periodogram of the RV residuals after removing the contribution of the transiting planet shows a significant peak at $\approx$0.1 $\hbox {d}^{-1}$. The FAP of this signal was assessed using a bootstrap randomization process. The RV values were randomly shuffled keeping the time stamps fixed and a GLS performed on the data. In 200,000 shuffles of the data there was no instance where the random periodogram had power higher than that of the data. The FAP is thus less than 5 $\times$ $10^{-6}$.

The 10 d signal may arise from stellar activity. The rotational modulation from surface structure can create RV variations at the rotation period of the star, $P_{rot}$, and its harmonics ($P_{rot}$/2, $P_{rot}$/3, etc.). To check for this we investigated quantities that measure the spectral line shapes since RV variations due to activity are often accompanied by spectral line shape changes. The full width at half maximum (FWHM) and span of the bisector (BIS) of the cross-correlation function are common tools for measuring line shape changes. The GLS periodograms of the nightly averages of these quantities show no significant peaks at or near a frequency of 0.1 $\hbox {d}^{-1}$ Indicators of chromospheric activity can also show evidence of the rotation period of the star and and the so-called S-index of the Ca II H & K lines is commonly used for this. Again, the GLS periodogram of the nightly average S-index values shows no significant signal at the RV period. Given the lack of variations in activity indicators at 10 d the most likely explanation for these RV variations is that they arise from the presence of an additional, non-transiting planet. The right panel of Fig. 19 shows the orbital fit to the 10 d RV variations, and the parameters are listed in Table 3.

**Table 3:** Orbital parameters from the FCO method.

Parameter	Planet b	Planet c
Period [days]	0.877171	9.93 ± 0.01
$\hbox {T}_{0}$ [BKJD]	2750.047572	$3031.4222 \pm 1.15$
*K* [m $\hbox {s}^{-1}$]	$4.54 \pm 0.88$	$5.94 \pm 0.47$
*e*	0.00 (fixed)	$0.13 \pm 0.07$
$\omega$ [deg]	90 (fixed)	18 ± 40

#### GP analyses of the activity indicators

We analyzed the activity indicator time series utilising a GP regression to find the GP hyperparameters that can characterise the stellar signal. We assume that each activity indicator time series can be described with the covariance function

1 $$\begin{aligned} \gamma _\textrm{1D,i,j} = A^2 \gamma _{i,j}, \end{aligned}$$

where A is an amplitude term, and

2 $$\begin{aligned} \gamma _{i,j} = \exp \left[ - \frac{\sin ^2[\pi (t_i - t_j)/P_\textrm{GP}]}{2 \lambda _\textrm{P}^2} - \frac{(t_i - t_j)^2}{2\lambda _\textrm{e}^2} \right] , \end{aligned}$$

is the Quasi-Periodic (QP) kernel where $P_\textrm{GP}$ is the GP period, $\lambda _p$ is the inverse of the harmonic complexity, and $\lambda _e$ is the long term evolution timescale. For the GP mean function, we model an offset term for each instrument. We also include a jitter term per instrument in the likelihood to penalise the imperfections of our model. We use the code pyaneti^51,52^ to create posterior of the QP kernel hyperparameters.

We ran a one-dimensional GP regression for the FWHM and S-index to characterise the scales of the stellar induced signals. We set wide uniform priors on the GP hyperparameters: $\lambda _\textrm{e} \in [1,500]$, $\lambda _\textrm{p} \in [0.1,5]$, and $P_\textrm{GP} \in [20,50]$. For the amplitude we also set uniform priors between 0 and the maximum peak-to-peak difference of each time series. We sample the parameter space with 250 walkers using the Markov chain Monte Carlo (MCMC) ensemble sampler algorithm implemented in pyaneti^51,53^. We created the posterior distributions with the last 5000 iterations of converged chains. We thinned our chains with a factor of 10 giving a distribution of 125 000 independent points for each sampled parameter.

The inferred values for the QP hyperparameters for the FWHM modelling are $\lambda _\textrm{e} = 383 _{ - 147 } ^ { + 85 }$ days, $\lambda _\textrm{p} = 3.5 _{ - 2.1 } ^ { + 3.6 }$, $P_\textrm{GP} = 34.8 _{ - 1.1 } ^ { + 1.0 }$ days. While the recovered QP hyperparameters from the $S_\textrm{HK}$ time series are $\lambda _\textrm{e} = 157 _{ - 50 } ^ { + 59 }$ days, $\lambda _\textrm{p} = 6.8 _{ - 2.7 } ^ { + 2.2 }$, $P_\textrm{GP} = 31.8 _{ - 2.9 } ^ { + 6.7 }$ days. We note that these result are not fully consistent between each other. This is likely caused by the sub-optimal sampling of the spectroscopic time series given that the main reasoning behind GP regression is to characterise the correlation between data. Implying that if the data sampling does not represent the time-scales that we want to characterise, this will lead to poorly constrained hyper-parameters. Notwithstanding, these results suggest that the signal has low harmonic complexity in both time series (i.e., it behaves similar to a sinusoidal). This is an indication that the underlying stellar signal in the RV time series will also have a low-harmonic complexity^52^.

#### Model selection

In order to account for the signals found in the frequency analysis and address their significance in the data, we carried out a model comparison scheme^54^. We used juliet^55^, a code which efficiently computes the Bayesian log-evidence of each tested model and explores the parameter space using the importance nested sampling included in MultiNest^139^ via the PyMultiNest package^140^. This method outperforms other samplers in choosing robustly the best model for those with 3 or less planets^141^. We considered a model to be moderately favored over another if the difference in its Bayesian log-evidence $\Delta \ln Z$ is greater than 2.5, while strongly favored if it is greater than five^56^.

We discuss several RV-only models assuming both circular and eccentric orbits with increasing number of Keplerian components. To model the RV variability associated to stellar activity we try to model it assuming a periodic and coherent behavior (with a sinusoid) and with a quasi-periodic GP kernel^142^, which has the form:

$$\begin{aligned} k_{i,j}(\tau ) = \frac{B}{2+C}e^{-\tau /L}\left[ \cos \left( \frac{2\pi \tau }{P_\text {rot}}\right) + (1+C)\right] \quad \end{aligned}$$

We placed Gaussian priors on the GP hyperparameters *L* and $P_\textrm{rot}$ determined by the posteriors derived from “training” the GP on the *K2* light curve. For convenience, we encoded the basic assumptions of each model into a condensed naming scheme, consisting of a series of numbers and letters. For example, a model consisting of a single Keplerian orbit is named “1c” if assumed to be circular and “1e” if allowed to be eccentric, a model consisting of a circular orbit for the first component and an eccentric orbit for the second is named “1c2e”, and models that include a GP noise model are indicated as such by the suffix “GP”. In all models we included jitter parameters ($\sigma$) and offsets ($\gamma$) for the two spectrographs.

Table 4 summarizes the results from the RV-only model comparison analysis. First, we confirm that the RV data is incompatible with the presence of only the USP transiting planet and that more signals are present in the data, in line with the results from our frequency analyses. We note that although the evidence favours a Keplerian orbit for the transiting planet in models with only one component, the derived eccentricity ($e=0.58\pm 0.07$) is extremely high for a USP planet and it is originated from not accounting for the remaining signals in the data and a poor sampling of the orbital phase close to superior conjunction. Therefore, we assume a circular orbit for the transiting planet in the subsequent analyses, which is a good assumption for USP planets due to their short tidal circularization timescales^143^. On the other hand, we assume an eccentric orbit for second component of the RV model although the Bayesian evidence for circular or eccentric orbits is approximately the same. For this second signal we derive a moderate eccentricity that improves the RMS of the fit and minimizes the jitter for both spectrographs.

The best-performing model consists of two Keplerian orbits and a GP noise model with a quasi-periodic kernel, whose hyperparameters come from the modeling of the *K2* light curve. Modelling the stellar component with a sinusoid, although an improvement with respect to models with only two components, is not enough to reproduce the change of stellar variability with time as seen in the stacked periodograms or the time series of the S-index.

**Table 4:** RV model selection via the Bayesian log-evidence $\ln Z$; the adopted model is in boldface.

Model	$N_\textrm{free}$	$\hbox {RMS}^a$	$\ln Z^b$
1c	7	6.27	− 377.11
1e	9	5.56	− 364.45
1c2c	10	4.35	− 348.50
1c2e	12	4.12	− 348.40
1c2cGP	17	2.84	− 322.36
**1c2eGP**	**19**	**2.79**	**− 319.71**
1c2c3c	13	3.93	− 342.47
1c2e3c	15	3.56	− 332.87

$^a$ Root mean square of the data minus the model.

$^b$ Bayesian evidence of the model

#### Doppler mass measurement

To obtain dynamical masses and orbital parameter estimates while accounting for stellar activity signals, we ran a multidimensional GP fit to our spectroscopic time series using the code pyaneti^51,52^. We adopted a 2-dimensional GP approach between the RVs and the $S_\textrm{HK}$. We used the $S_\textrm{HK}$as has been shown to be a good tracer of the stellar activity in spectroscopic time series^58,59^.

We created our 2-dimensional GP regression by assuming that our RV and $S_\textrm{HK}$ time series can be modelled as

3 $$\begin{gathered} \Delta {\text{RV}} = V_{c} G(t) + V_{r} \dot{G}(t), \hfill \\ \Delta {\text{S-index}} = S_{c} G(t), \hfill \\ \end{gathered}$$

where *G*(*t*) is a latent (unobserved) variable, which can be thought as a function representing the projected area of the visible stellar disc that is covered in active regions at a given time, and $\dot{G}(t)$ corresponds to its time derivative^52,57^. We created the multidimensional covariance matrix using the QP kernel given in Eq.2 as implemented in pyaneti. For the GP regression, as a mean function for the RVs ($m_\textrm{RV}$) we use the sum of two Keplerian curves, including offsets. The mean function for the $S_\textrm{HK}$ time series is just an offset for each instrument. We also include a white noise jitter term in the likelihood for each instrument and time series to penalise the imperfections of the model.

We then sampled the posterior via an MCMC sampling using the same configuration as in our GP analysis of the activity indicators. We set uniform priors for the hyperparameters $\lambda _e$, $\lambda _p$, and $P_\textrm{GP}$. We set Gaussian priors on the orbital epehemerides of both planets based on the transit analysis. We assume the orbit of planet b to be circular, and for planet c we sample for an eccentricity and angle of periastron. The rest of orbital and planetary parameters were treated with wide uniform priors.

Figure 3 shows the modelling of our RV and $S_\textrm{HK}$ time series. For planets b and c we detect Doppler semi-amplitudes of $5.19 \pm 0.51$ $\textrm{m}\,\textrm{s}^{-1}$ and $4.77 \pm 0.71$ $\textrm{m}\,\textrm{s}^{-1}$, respectively. This translates into planetary masses of $5.8 \pm 0.8 M_\oplus$ and $11.8 \pm 2.2 M_\oplus$ for for planets b and c. For planet c we recovered a small eccentricity of $0.11_{-0.8}^{+0.1}$. The full set of parameter estimates are presented in Table 1, and the RV data and model constraints are shown in Fig. 3.

The inferred values for the QP hyperparameters are $\lambda _\textrm{e} = 206_{-51}^{+71}$ days, $\lambda _\textrm{p} = 7.5 _{ - 2.2 } ^ { + 1.8 }$, $P_\textrm{GP} = 37.6 _{ - 1.1 } ^ { + 0.7 }$ days. We note that the recovered period is consistent with the period found in the $\ell _1$ periodogram on the RV data. This suggest that in this analysis, The RVs dominate the determination of the QP period. The relatively high value of $\lambda _\textrm{p}$ implies that the stellar signal has a low harmonic complexity, and thus appears nearly sinusoidal (see Fig. 3). This behavior is also supported by the relatively large value of $\lambda _\textrm{e}$, which implies that the active regions on the stellar surface can survive several rotational periods.

### Stability and migration via tidal dissipation

**Fig. 21:**
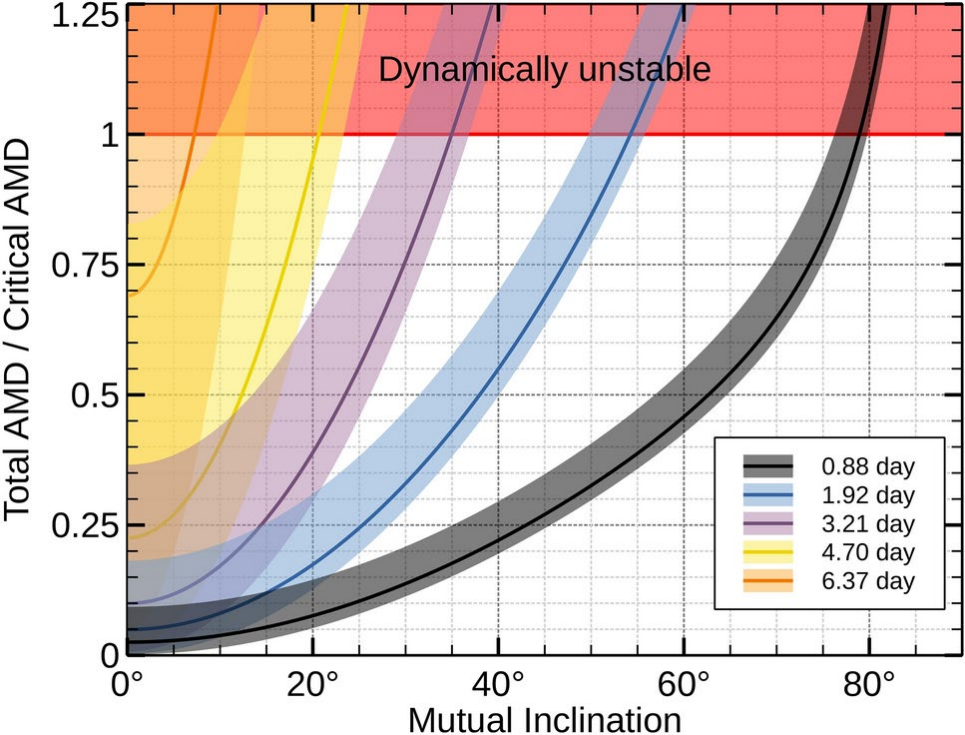
Critical angular momentum deficit as a function of mutual inclination, for different values of the period of K2-360  b. The red line is the threshold above which the system is unstable. The shaded region indicates the uncertainty in the AMD due to the uncertainty in the eccentricity of K2-360  c.

USPs are thought to have migrated to their current location from more distant orbits through either disk migration or tidal dissipation^64–66,74,75^. In the latter case, it requires some mechanism to pump the eccentricity of the inner planet. The detection of K2-360  c at a period of 9.8 days poses constraints on the upper limit of the hypothetical initial period of K2-360 b, because of dynamical stability. This upper limit depends also on the mutual inclination between the two planets, with low-inclination orbits able to have longer periods before being dynamically unstable. Constraining the available parameter space for the initial orbit of K2-360 b is crucial for understanding its possible migration mechanism.

We use the angular momentum deficit (AMD) stability criterion to obtain an upper limit **on** the mutual inclination between the inner and the outer planet^60,61,63^. In Fig. 21 we plot the ratio between the system AMD and the critical AMD as a function of mutual inclination $i_\textrm{mut}$ for several value**s** of the period of K2-360 b. Systems with an AMD ratio above 1 are dynamically unstable. In the present configuration, the maximum allowed **mutual** inclination for the system to be stable is about $i=80^\circ$. In the above analysis we adopt a mass of 15.23 $M_{\oplus }$ for K2-360  c.

The orbital configuration of K2-360 provides an excellent opportunity to test migration theories of USPs. In particular, the presence of a massive, inclined outer planet may induce the secular excitation of the eccentricity of the USP progenitor. Mutual inclinations higher than ${\sim }40^\circ$ may even induce secular forcing in the form of ZKL oscillations^68–70^. In this latter scenario, the outer, inclined planet would increase the eccentricity of the inner one, triggering high-eccentricity tidal migration.

It is possible to obtain an approximate analytic estimate for the migration timescale of a planet due to secular forcing from an outer one. Following^64,67^, and adopting a tidal quality factor $Q/k = 100$, we find a migration timescale of

4 $$\begin{aligned} \tau _\textrm{m} \approx 2\times 10^9 \,\textrm{yr} \left( \frac{Q/k}{100}\right) \left( \frac{1.57 \,{R}_\oplus }{R_b}\right) ^5 \left( \frac{M_b}{7.67 \,{M}_\oplus }\right) \left( \frac{P_b}{0.878 \,\mathrm d}\right) ^{-7/3} \left( \frac{P_c}{9.796 \,\mathrm d}\right) ^{16/3} \left( \frac{15.23 \,{M}_\oplus }{M_c}\right) ^2 \left( \frac{0.11}{e_c}\right) ^2\,, \end{aligned}$$

which indicates that the K2-360 b should be able to migrate to its current location due to the forcing of K2-360  c.

To test this hypothesis, we run a grid of simulations with the secular evolution code okinami, which solves the secular equations of motions of the three-body Hamiltonian developed at the octupole order in Delaunay orbital elements^71,72^. We also include tidal dissipation of the inner orbit due **to** equilibrium tides using a weak friction model^144^, and apsidal precession due to the tidal bulges and general relativity^145^. The equation**s** of motions are integrated with a 7th order Runge-Kutta integrator with adaptive timestep.

We uniformly sample the initial period of planet b between 1 and 4.7 days. K2-360  b might have had a gaseous envelope in the past, and subsequently lost it via tidal heating and photoevaporation^104^. To take this into account, we choose a radius of 2$R_{\oplus }$ for K2-360  b. We adopt a mass of 7.7 $M_{\oplus }$ and 15.2 $M_{\oplus }$ for the inner and outer planets, respectively. The mutual inclination between the two planets is sampled uniformly between $10^\circ$ and $80^\circ$. We ran a total of 4096 simulations, assuming a tidal time-lag of $0.66 \mathrm \, s$ and an apsidal motion constant $k_2 = 0.1$. This corresponds to a modified tidal quality factor of $Q^{\prime } = 200$ for a planet at 0.88 days, consistent with the expected composition of K2-360 b. Each realization is run for 6 Gyr.

Despite the large uncertainties in tidal parameters, this grid of simulations can provide insights on the initial configuration of the system. Figure 4 shows the final distribution of the orbital period of the inner planet at the end of the simulations. A consistent fraction of the realizations ended up with planet b on an orbit with a period less than one day. The migration occurs most often when the two planets have large mutual inclinations, where the ZKL mechanism is most efficient. On the other hand, a small fraction of planets can migrate at low mutual inclinations. After the migration has occurred, the distribution of mutual inclinations peaks at 20–40 degrees. While the ZKL mechanism requires substantial initial mutual inclinations between the two inner planets, stellar flybys, disk warping or planet-planet interactions could produce the required misalignment^146–148^.

We have performed additional N-body simulations, including equilibrium tides, spins and first-order post-Newtonian corrections with the regularized few-body code tsunami^149,150^. In these simulations, we initialized K2-360 b with an obliquity of 15 degrees, and placed the outer planet on an 20 degrees inclined orbit. We found that the planet quickly settles into a Cassini state with an obliquity of about 10 degrees and subsequently obliquity tides drive an orbital decay of 2.6–$0.5 {\,\mathrm d / Gyr}$ for planets with an initial period of 3–6 days. Such simulations are too computationally expensive to be run until K2-360 b reaches its observed orbit, but the measured initial decay rate is consistent with the following analytic estimate:

5 $$\begin{aligned} t_\textrm{m} \approx 2\times 10^9 \,\textrm{yr} \left( \frac{Q/k}{275}\right) \left( \frac{1.57 \,{R}_\oplus }{R_b}\right) ^5 \left( \frac{M_b}{7.67 \,{M}_\oplus }\right) \left( \frac{P_b}{4 \,\mathrm d}\right) ^{13/3} \left( \frac{F(e_b=0.05, \epsilon _b=15^\circ )}{22}\right) \,, \end{aligned}$$

where $F(e_b, \epsilon )$ is a known function of eccentricity and obliquity^66^. Our results show that obliquity tides are also a viable pathway for the formation of K2-360 b.

### Interior structure

**Fig. 22:**
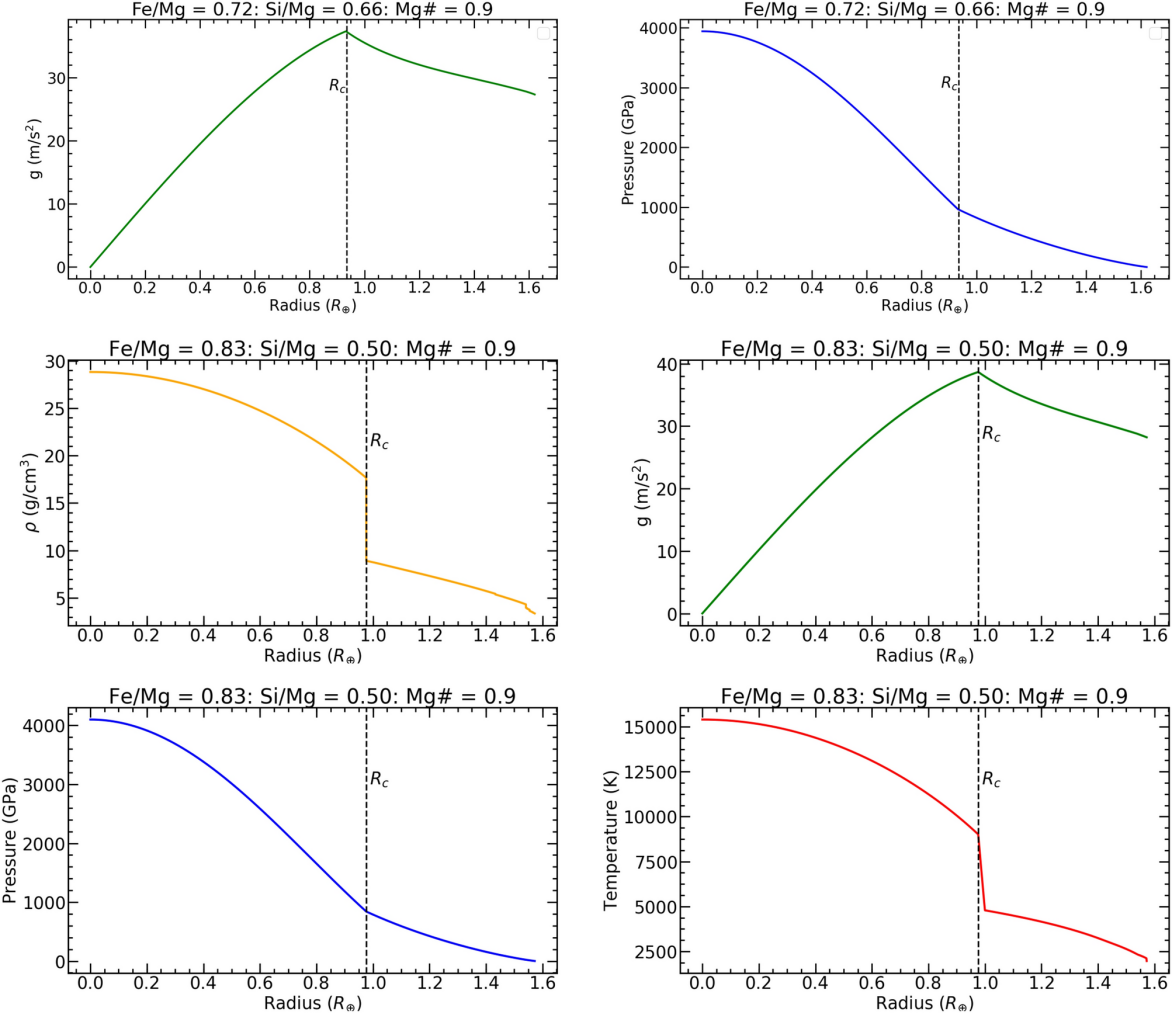
The density, gravity, pressure and temperature (Y-axis) profiles against planetary radius (X-axis) for different interior conditions of K2-360  b. The top two panels show the gravity and pressure profiles at the observed stellar abundances. The bottom four panels show the density, gravity, pressure and temperature profiles for K2-360  b if the Fe and Mg abundances are higher than what was measured.

Our interior structure model for K2-360  b assumes spherical symmetry for the planet^78^. We also assumed that the planet is differentiated into convective, concentric layers of core, lower mantle, upper mantle and crust. Each layer was given an adiabatic temperature profile. The pressure (*P*), gravity (*g*) and density ($\rho$) profiles were evaluated with a set of differential equations that were integrated radially outwards from the centre of the planet to the surface. The density at a given depth was found with an Equation of state (EoS) specific to that particular layer and its composition. The core was modelled with a temperature-dependent EoS^81^ and assumed to be composed of iron (Fe), in a hexagonal close-packed (hcp) structure^151^. , We assumed a standard EoS for the density of mantle minerals^79,80^.

We assumed that the elemental abundances between the planet and host star are identical^82–85^. The spectroscopic parameters of K2-360 resulted in Fe/Mg, Si/Mg and Ca/Mg ratios of 0.72, 0.66, and 0.067, respectively, for K2-360 b. Since there was constraint on the Al/Mg abundance, a ratio of 0.083 was assumed, similar to that of the sun. An Mg# of 0.9 was assumed for the mantle of K2-360  b^85,152^. The lower mantle was assumed to include $\hbox {MgSiO}_{3}$, $\hbox {FeSiO}_{3}$, $\hbox {CaSiO}_{3}$, $\hbox {Al}_{2}\hbox {O}_{3}$, MgO and FeO^80,153–155^. The upper mantle and crust was assumed to be primarily composed of $\hbox {Mg}_{2}\hbox {SiO}_{4}$ and $\hbox {Fe}_{2}\hbox {SiO}_{4}$^156–158^. The Fe/Mg ratio of the planet was used to determine the CMF.

An optimal CMF of 48 percent was found for K2-360  b. With a CMF of 48 per cent, the best resultant model had a radius of 1.6$R_{\oplus }$ at a mass of 7.7$M_{\oplus }$, which is within the error bars of the measured mass and radius of the planet. A smaller radius at the same mass could be found for a higher CMF, but that led to a Fe/Mg ratio that is closer to 0.80 than the 0.72 that was determined based on the stellar abundances. We did not consider a liquid Iron portion in the core because of the high mass of this planet^159,160^. The CMF of this planet (48%) is much higher than that of the Earth (32%), yet less than that of Mercury ($\sim$70%), making this more similar to a Super-Earth than a Super-Mercury. A majority of *Kepler* USP planets discovered had radii between 1$R_{\oplus }$ and 2$R_{\oplus }$ and masses ranging from 2$M_{\oplus }$ to 8$M_{\oplus }$^161^. This planet falls within the parameter range of previously validated USPs. We ran another model with different elemental abundances to explore an alternative interior structure. We input values of Fe/Mg = 0.84 and Si/Mg = 0.5, to make it an Fe and Mg rich planet. The bottom four panels of Fig. 22 show the internal radial profiles with these alternative model parameters. With this model we get a CMF of 57% and a radius of 1.58$R_{\oplus }$ at 7.8 $M_{\oplus }$, making it closer to the observed planet radius while still being in good agreement with the observed planet mass. Given the data, a CMF between 48% and 57% seems likely for K2-360  b.

## Data Availability

The *K2* and *TESS* data used in this work are publicly available in the Mikulski Archive for Space Telescopes (MAST). The HARPS data used in this work are publicly available in the European Southern Observatory (ESO) archive. The HARPS-N data used in this work are publicly available in the Telescopio Nazionale Galileo (TNG) archive. The NESSI data used in this work are publicly available via the Exoplanet Follow-up Observing Program (ExoFOP) operated by the California Institute of Technology. Other data products used in this study are available from the corresponding author on reasonable request.
